# Influence of reward-related genetic variants on BMI and predisposition to obesity: Systematic review and meta-analysis

**DOI:** 10.1590/1678-4685-GMB-2025-0216

**Published:** 2026-05-22

**Authors:** Bernardo Perin Cima, Mariana Meira Scudeler, Juliana Dal-Ri Lindenau, Yara Costa Netto Muniz

**Affiliations:** 1 Universidade Federal de Santa Catarina, Laboratório de Polimorfismos Genéticos (Lapoge), Florianópolis, SC, Brazil.

**Keywords:** BMI, obesity, brain reward cascade, neurogenetics

## Abstract

Obesity results from a chronic imbalance between caloric intake and energy expenditure, leading to the accumulation of excess body fat. This imbalance is influenced by endocrine, molecular, genetic, demographic, environmental, and lifestyle factors, with the brain reward cascade emerging as a key overlapping pathway. This study hypothesizes that cascade dysregulation leads to reward deficiency, causing stronger cravings and overeating. This study investigates the contribution of genetic variants in the brain reward cascade to body mass index (BMI), overweight, and obesity. A systematic search was conducted across five databases. Eligible studies evaluated associations between variants in reward-related genes (*COMT*, *DRD2*, *DRD4*, *MAO-A*, *SLC6A3*, *SLC6A4*) and BMI or obesity. In total, 117 studies were included in the systematic review, and 31 provided sufficient data for meta-analysis. Eighteen variants showed significant associations with BMI, with the strongest evidence observed for the Val158Met (*COMT*), Taq1A (*DRD2*), 48 bp VNTR (*DRD4*), 40 bp VNTR (*SLC6A3*) and 5-HTTLPR (*SLC6A4*). Meta-analyses of Taq1A showed a significant association of the A1 allele with obesity. This study highlights consistent associations between genetic variants in the reward cascade and susceptibility to weight gain, providing a foundation for more precise prevention and personalized obesity management.

## Introduction

Obesity is a chronic disease characterized by excessive fat accumulation, affecting an estimated 890 million adults worldwide. Obesity is diagnosed using the body mass index (BMI), calculated as weight in kilograms divided by height in meters squared (kg/m²) (WHO, 2025). BMI is an indirect measure of adiposity and related risks, suitable for population-level assessments and screening rather than individual diagnosis (Rubino et al., 2025).

Obesity results from a chronic imbalance between caloric intake and energy expenditure, leading to the accumulation of excess body fat (Hill et al., 2012). This imbalance reflects a complex interplay of endocrine, molecular, genetic, demographic, environmental, and lifestyle factors, with the brain reward cascade representing a key area of overlap (Gold et al., 2025). Dysregulation in this cascade can lead to a reward deficiency where individuals may have stronger cravings and tend to overeat to compensate for reduced reward sensitivity (Volkow et al., 2011).

The brain reward cascade begins when external stimuli activate serotonergic neurons in the hypothalamus, which release serotonin that subsequently stimulates enkephalinergic neurons. The enkephalins inhibit GABA, which normally suppresses dopamine activity. With this inhibition reduced, dopamine is released and activates D2 receptors in the nucleus accumbens, generating the sensation of reward (Blum et al., 2000). 

Several genetic variants affect the brain reward cascade. The reward-related genes most studied in relation to obesity are *COMT*, *DRD2*, *DRD4*, *MAO-A*, *SLC6A3*, and *SLC6A4*. These genes encode key proteins that regulate dopaminergic and serotonergic signaling. *COMT* and *MAO-A* encode enzymes involved in neurotransmitter degradation (Wilson et al., 1984; Shih, 1991), *DRD2* and *DRD4* encode dopamine receptors (Grandy et al., 1989; Chen et al., 1999), while *SLC6A3* and *SLC6A4* encode transporters responsible for dopamine (Kawarai et al., 1997) and serotonin reuptake (Pinto et al., 2008).

While the involvement of these genes is well-documented in the context of substance-related and behavioral addictions, such as alcoholism, smoking and gambling, their role in eating behavior, weight regulation, and obesity is not yet fully understood (Blum et al., 2014). This gap in knowledge is particularly relevant given the high costs of obesity treatment for public health, which highlights the urgent need for preventive strategies (Cawley et al., 2021). Pharmacological options, such as GLP-1 receptor agonists, can induce weight loss but are associated with adverse effects, including nausea, vomiting, diarrhea, pancreatitis, and gallbladder disease (Wilding et al., 2021), and may disrupt dopaminergic signaling, contributing to depression and suicidality (Blum *et al*., 2025a). In this context, understanding a patient’s genotype offers an alternative pathway, enabling the identification of a “pre-addiction” phenotype (Blum *et al*., 2025b). Such knowledge can support the prescription of personalized treatment or prevention strategies, reducing reliance on pharmacological approaches and supporting personalized obesity management (Blum *et al.*, 2018; Loos and Yeo, 2022).

Despite increasing interest in the contribution of reward-related genes to body weight regulation, evidence linking these variants to BMI and obesity remains limited and fragmented. This review systematically synthesizes and quantitatively analyzes available studies to map genetic variants within the brain reward system associated with BMI, overweight and obesity. 

## Material and Methods

Systematic reviews provide authors with the opportunity to determine the extent of human knowledge on specific topics and to establish priorities for future research (Lasserson *et al*., 2021). This section describes the steps for conducting the systematic review and meta-analysis.

### Research question

The central question guiding this systematic review is: Which variants in the *COMT*, *DRD2*, *DRD4*, *MAO-A*, *SLC6A3*, and *SLC6A4* genes significantly influence increased BMI or predisposition to obesity, and what are the qualitative and quantitative effects of these variants?

### Search strategy

The search strategy was developed by combining the names and variations of the genes of interest (*COMT*, *DRD2*, *DRD4*, *MAO-A*, *SLC6A3* and *SLC6A4*) with the terms “BMI,” “Overweight,” and “Obesity,” as well as their synonyms and database-specific entry terms. The full search strategy is available in Table S1.

Searches were conducted in July 2025 across five databases: PubMed, Scopus, Embase, LILACS, and Web of Science. Additionally, the first 100 results from Google Scholar were screened, and reference lists of included studies were manually searched to identify any additional relevant publications.

The eligibility criteria were based on the PICO framework: Population, Intervention, Comparison, and Outcome (Santos et al., 2007). Studies were included if they assessed the association between genetic variants of the selected genes and BMI, overweight or obesity in human participants. There was no restriction regarding the year of publication.

Initial screening of articles was performed based on titles and abstracts. Full-text screening followed for those that met the inclusion criteria. Two independent reviewers conducted the selection process to ensure reliability.

The results of the article collection, selection, and exclusion process are presented in a flowchart. 

### Data extraction

Data was extracted independently by two reviewers to minimize the risk of bias and errors. Qualitative data were categorized according to whether they reported significant associations with BMI, overweight, or obesity. Results were divided by genes and were discussed in terms of the possible biological mechanisms through which the variants may influence BMI. Quantitative data was subjected to meta-analyses.

### Methodological quality assessment of the included studies

The methodological quality of the included studies was assessed using the Q-Genie tool, specifically developed and validated for genetic association studies. This instrument allows for a systematic evaluation of key methodological aspects relevant to genetic research, including genotyping procedures, Hardy-Weinberg equilibrium, population stratification, and reporting of genetic variants. Each item is scored on a scale from 1 (Poor) to 7 (Excellent), and the total score classifies study quality as follows: for studies with control groups, scores ≤35 indicate poor quality, >35 and ≤45 indicate moderate quality, and >45 indicate good quality; for studies without control groups, scores ≤32 indicate poor quality, >32 and ≤40 indicate moderate quality, and >40 indicate good quality (Sohani et al., 2015). 

### Statistical analysis

Studies with equivalent data were pooled, and the effects of functional alleles were assessed through meta-analyses, generating a more robust estimate of effect size (Deeks et al., 2024). Equivalent data refers to studies that reported genotypic means divided by the same genotypes, or allelic/genotypic frequencies categorized using the same BMI classification. 

All analyses were conducted using the R Statistical Software with the “meta” package, employing the “metabin” and “metacont” functions (Schwarzer, 2007; Balduzzi et al., 2019).

Two types of meta-analysis were conducted: 1. Binary data meta-analysis, based on allele and/or genotype frequencies according to BMI categories; 2. Continuous data meta-analysis, based on mean BMI values across genotypic groups.

For binary data, the metabin function was used. Participants were grouped by BMI category following WHO (2025) definitions: healthy weight (BMI 18.5-24.9), overweight (BMI 25-29.9), and obesity (BMI ≥ 30). The effect measure used was the Odds Ratio. Associations between allele/genotype frequencies and BMI categories were estimated using pooled odds ratios with 95% confidence intervals, applying both fixed-effect and random-effects models. The fixed-effect model employed the Mantel-Haenszel method (Mantel and Haenszel, 1959; Robins et al., 1986) and the random-effect model used the inverse-variance weighting method. Heterogeneity among studies was assessed using tau-squared (τ²) calculated with the Paule-Mandel method (Paule and Mandel, 1982). I² values of 25%, 50%, and 75% were considered indicative of low, moderate, and high heterogeneity, respectively. For the random-effects model, the Knapp-Hartung adjustment was applied to reduce the risk of a false positive result (Knapp and Hartung, 2003). Although results from both models are reported, we prioritized the random-effects estimates. However, in cases of low heterogeneity, fixed-effect results may also be considered reliable

Continuous data analyses were performed using BMI means by genotype, regardless of BMI classification. The effect size was expressed as the standardized mean difference, measured through Hedges’ g (Hedges and Olkin, 1985). Variance estimation was conducted using the Restricted Maximum Likelihood (REML) method (Viechtbauer et al., 2015), and the Knapp-Hartung adjustment was again applied for improved statistical reliability. Alleles were grouped as follows: homozygous for the functional allele + heterozygous vs. homozygous for the opposite allele. The results are presented in forest plots. 

Meta-analyses yielding significant results had the quality of the generated evidence assessed using the Venice Criteria, which consider the total number of patients, heterogeneity, result consistency, Hardy-Weinberg equilibrium, and the methodological quality of the included studies. This is the most recommended methodology for genetic association studies, as it is based not on the study design but on the robustness of the association (Ioannidis et al., 2008). 

This systematic review was conducted in accordance with the PRISMA (Preferred Reporting Items for Systematic Reviews and Meta-Analyses) and Cochrane guidelines (Higgins *et al*., 2022). The PRISMA checklist is available as Table S2. This review was registered with PROSPERO (International Prospective Register of Systematic Reviews) under ID CRD420250651974.

## Results

### Study selection, characterization and association with BMI, overweight or obesity

**Figure 1 -:**
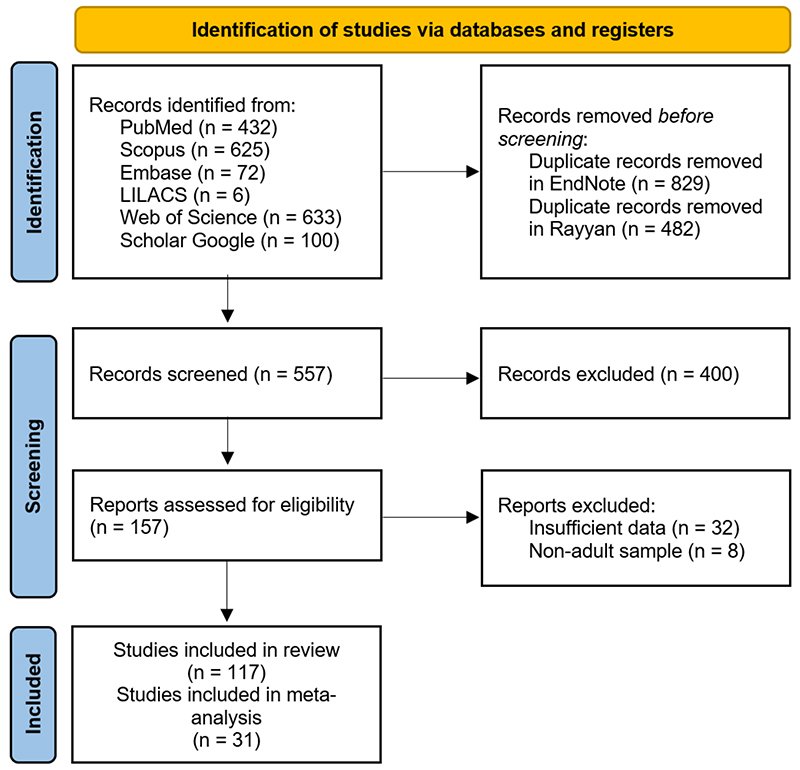
Flow diagram of the article screening process using PRISMA guidelines. Adapted from: Page et al. (2021).

A total of 1,868 articles were identified through the search mechanisms, of which 117 were included in the systematic review and 31 in the meta-analyses. The complete flowchart of the article selection process for the systematic review is shown in Figure 1.

**Figure 2 -:**
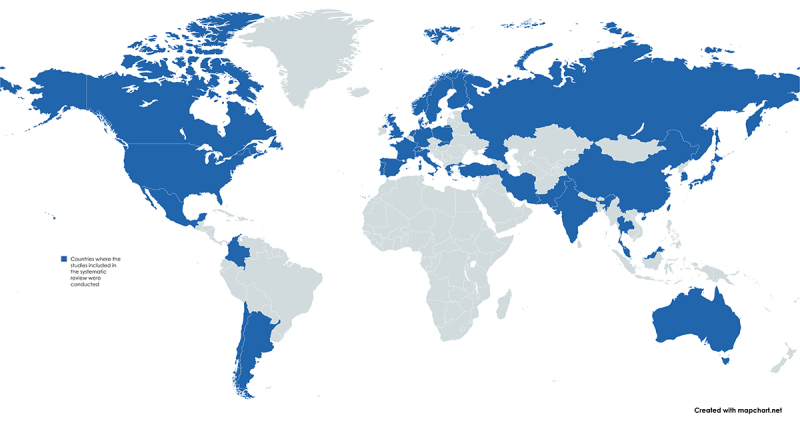
Geographic distribution of the 117 studies included in the systematic review. The articles included in the review originate from 34 countries, which are highlighted in blue on the map. No studies from the African continent met the inclusion criteria for this review.

The included studies were published between 1993 and 2025, originating from 34 countries. No studies from Africa or Brazil met the inclusion criteria. The geographic distribution of the included studies is shown in Figure 2.

Table 1 presents the 18 variants that showed a significant association with BMI. A complete list of variants without association is provided in Table S3. The full list of the 117 articles included is available in Table S4, and the methodological quality assessment of these studies is presented in Table S5.

**Table 1 -:** Summary of variants significantly associated with BMI or obesity.

Gene	Variant	Study	Association
***COMT***	His62His (rs4633)	Yang et al., 2014	rs4633 showed significant association signals with BMI
	Leu136Leu (rs4818)	Wang et al., 2007	The G allele was more frequent in individuals with obesity than in normal-weight controls
	rs2020917	Mehri et al., 2019	T allele was associated with higher BMI
	Val158Met (rs4680)	Hong et al., 2003	In premenopausal subjects, the low-activity allele was associated with greater BMI
		Cribb et al., 2011	The high-activity allele was much more frequent in the higher BMI group
		Valomon et al., 2014	BMI was lower in Val/Met heterozygotes compared with Val/Val homozygotes
***DRD2***	Taq1A (rs1800497)	Thomas et al., 2001	A1 allele was more frequent in individuals with obesity
		Morton et al., 2006	Taq1A T allele was more frequent in individuals with obesity than in normal-weight individuals
		Chen et al., 2007	Prevalence of A1 allele was significantly higher in individuals with obesity than in individuals with normal weight
		Chen et al., 2012a	A1 allele was much more frequent in individuals with overweight or obesity
		Winkler et al., 2012	A1+ genotypes were associated with higher body mass index
		Thaler et al., 2012	Combination of low-function DRD2 T allele and DAT 10/10 genotype was associated with higher BMI compared to those with the 10/10 genotype but without the DRD2 T allele
		Carpenter et al., 2013	Mean BMI was significantly higher among participants carrying one or more copies of A1 allele compared to participants with no A1 alleles
		Yadav et al., 2016	Presence of homozygous A1A1 genotype was highest in the obesity category compared to underweight and normal categories
		Aliasghari et al., 2021	T allele was more frequent in individuals with obesity/overweight than in normal-weight individuals
		Hidalgo Vira et al., 2023	Homozygous A1/A1 carriers showed higher BMI compared with A1/A2 and A2/A2 carriers in the group with obesity
	Taq1B (rs1079597)	Spitz et al., 2000	B1 allele was more frequent in individuals with obesity
	Ser311Cys (rs1801028)	Chen et al., 2012b	Frequencies of Ser/Cys genotype and Cys allele were higher in individuals with obesity compared to controls
	C957T (rs6277)	Kvaløy et al., 2015	C allele displayed a protective effect against overweight development
	IVS6-83 (rs1076560)	Morton et al., 2006	The T allele was more frequent in individuals with obesity than in normal-weight individuals
	−141C Ins/Del (rs1799732)	Aliasghari et al., 2021	The Del allele was more frequent in individuals with overweight or obesity than in normal-weight individuals
		Arrue et al., 2023	Homozygous Ins/Ins patients had a higher frequency of obesity-related BMI than patients carrying the Del allele
	(rs2511521)	Pedram et al., 2017	G allele had higher frequencies in the control group
***DRD4***	48 bp VNTR	Eisenberg et al., 2008	BMI was higher in individuals with one or two 7R alleles in a nomadic population
		Levitan et al., 2010	7R carriers born in the fall had a greater BMI
		Sikora et al., 2013	Women with the 2R/2R genotype had the lowest BMI, while those with 7R/7R had the highest
		González-Giraldo *et al.*, 2017	4/4 genotype was associated with lower mean BMI
	-521C/T (rs1800955)	Munafò et al., 2006	Individuals with CC genotype showed greater BMI
***MAO-A***	30 bp VNTR	Ducci et al., 2006	Individuals with the low-activity allele showed greater BMI
		Need et al., 2006	Group with obesity showed an excess of the low-activity genotype
***SLC6A3***	40 bp VNTR (rs28363170)	Epstein et al., 2002	Genotype 10/10 in African Americans was more frequent in individuals with obesity
		Azzato et al., 2009	Individuals with obesity were less likely to carry the 3′ VNTR variant allele with 9 copies
		Sikora et al., 2013	Mean BMI was higher for the SS genotype than for combined LL and LS genotypes
		Bieliński et al., 2017	Homozygotes for the 9R allele were associated with the highest BMI
		González-Giraldo *et al.*, 2017	10/10 genotype was associated with higher mean BMI
	1215A>G (Ex9-55)	Azzato et al., 2009	Underweight individuals were significantly more likely to carry the G allele for SLC6A3
	G2319A (rs27072)	Pavlova et al., 2019	Carriers of the G allele had significantly higher BMI compared with homozygous AA carriers
***SLC6A4***	5-HTTLPR	Sookoian et al., 2008	Obesity was higher among individuals with the SS allele.
		Tsuboi et al., 2011	Subjects with the long allele showed higher BMI
		Peralta-Leal et al., 2012	Individuals with the long allele were more frequently classified as having overweight or obesity
		Markus et al., 2012	BMI increased significantly with presence of the S allele
		Borkowska et al., 2015	Patients with obesity carrying the L/L genotype had significantly higher BMI.
		Dias *et al.*, 2015	Overweight/obesity was significantly associated with the L allele
		Asadzadeh et al., 2019	Individuals without the long allele (SS) were at risk for higher levels of obesity
		Galaviz-Hernández et al., 2020	L allele was a risk factor for developing obesity in Mexican women with high NA ancestry

Some genes and variants are referred to by different names in the studies but have been standardized for this table.

### Meta-analysis

In total, 31 studies were included in the meta-analyses. It was possible to conduct meta-analyses using both binary and continuous data for the Taq1A (*ANKK1*/*DRD2*) and 5-HTTLPR (*SLC6A4*) variants. Meta-analyses could not be performed for variants in the *COMT*, *DRD4*, *MAO-A*, and *SLC6A3* genes due to the absence of studies and comparable data across the included articles.

**Figure 3 -:**
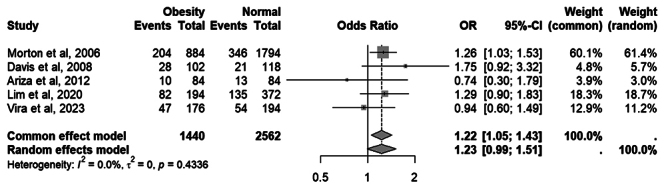
Meta-analysis of studies comparing the frequency of the A1 allele of the Taq1A variant in the *DRD2* gene between the obesity group (BMI ≥ 30) and the normal-weight group (BMI < 25). “Events” refers to the number of A1 alleles and “Total” refers to the number of alleles. Odds Ratio (OR) and 95% confidence intervals (CI) values are presented for fixed and random effect models.

When comparing the frequency of the Taq1A A1 allele across studies by dividing participants into the group with obesity (BMI ≥ 30) and the normal-weight group (BMI < 25) using a random-effects model, the analysis was borderline significant (OR = 1.23; 95% CI = 0.99-1.51; *p* = 0.0562). However, considering that study heterogeneity was null (I² = 0; t² = 0) and assuming a fixed-effect model, the A1 allele was significantly more frequent in the group with obesity compared to the normal-weight group (OR = 1.22; 95% CI = 1.05-1.43; *p* = 0.0096). The significant result obtained in the fixed-effect model was assessed using the Venice Criteria. Considering that the analysis involved more than 1,000 patients (A), showed low heterogeneity but inconsistent results (B), and that the quality of the studies included was considered high (A), the final evidence quality rating was ABA, classified as strong evidence (Ioannidis et al., 2008). The complete forest plot is available in Figure 3. To assess the influence of outliers in the sample, the analysis was repeated after removing Ariza *et al*. (2012). The significance of the results remained unchanged (random-effects model: OR = 1.24; 95% CI = 0.99-1.57; *p* = 0.0570).

**Figure 4 -:**
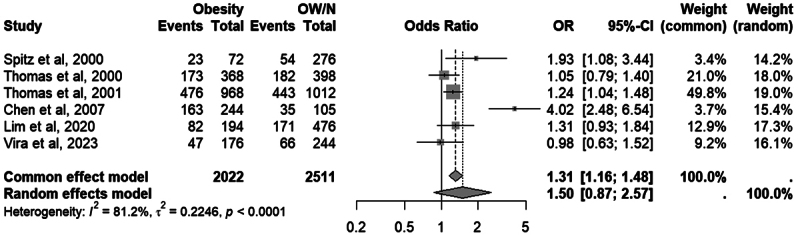
Meta-analysis of studies comparing the frequency of the A1 allele of the Taq1A variant in the DRD2 gene between the group with obesity (BMI ≥ 30) and the group without obesity (BMI < 30). “OW/N” refers to the group with overweight or normal weight (without obesity), “Events” refers to the number of A1 alleles and “Total” refers to the number of alleles. Odds Ratio (OR) and 95% confidence intervals (CI) values are presented for fixed and random effect models.

When comparing the group with obesity (BMI ≥ 30) and the group without obesity (BMI < 30), six studies could be included in the analysis, with two showing individually significant results. Under a fixed-effects model, the association was statistically significant (OR = 1.31; 95% CI = 1.16-1.48; *p* < 0.0001). However, due to the high heterogeneity observed (I² = 81.2%; t² = 0.2246, *p* < 0.0001), the random-effects model showed no significant overall association (OR = 1.50; 95% CI = 0.87-2.57; *p* = 0.1138). The complete forest plot is presented in Figure 4. 

**Figure 5 -:**
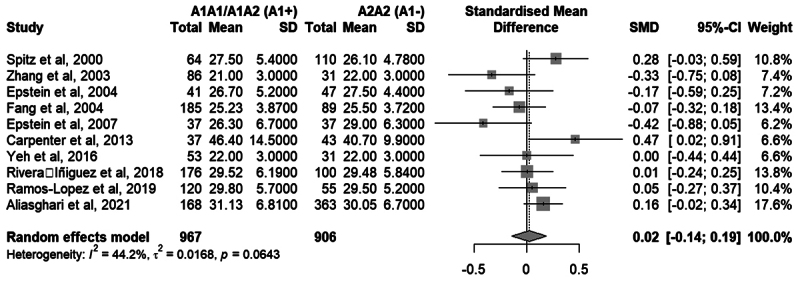
Meta-analysis of studies comparing the genotypic means of the Taq1A variant of *DRD2* in relation to the A1A1/A1A2 and A2A2 groups. “Total” refers to the number of individuals with the indicated genotypes. “Mean” refers to the mean BMI of the group. “SD” refers to the standard deviation. Standardized Mean Difference and 95% confidence intervals (CI) values are presented for random effect models.

Ten studies were included in the meta-analysis of the Taq1A variant, totaling 1,873 participants. The random-effects model, with Knapp-Hartung adjustment, indicated a standardized mean difference (SMD) of 0.0235 (95% CI: -0.1426 to 0.1896), with no statistical significance (*p* = 0.7561). Figure 5 presents the complete results of the genotype-based meta-analysis for the Taq1A genotypes.

**Figure 6 -:**
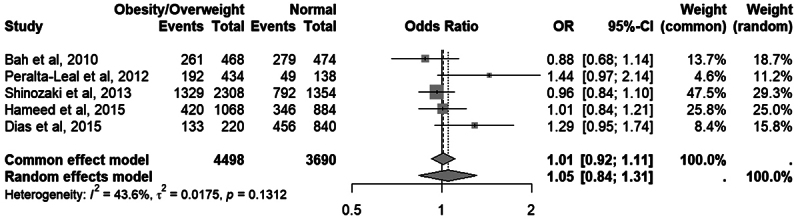
Meta-analysis comparing the frequency of the long allele of the 5-HTTLPR variant in the *SLC6A4* gene between individuals with overweight/obesity (BMI ≥ 25) and normal-weight individuals (BMI < 25). “Events” refers to the number of long alleles and “Total” refers to the number of alleles. Odds Ratio (OR) and 95% confidence intervals (CI) values are presented for fixed and random effect models.

In five studies analyzing the 5-HTTLPR variant, moderate heterogeneity was observed (I² = 43.6%), but the random-effects model showed no significant association with overweight/obesity (OR = 1.05; 95% CI: 0.84-1.31; *p* = 0.584). The complete forest plot is shown in Figure 6.

**Figure 7 -:**
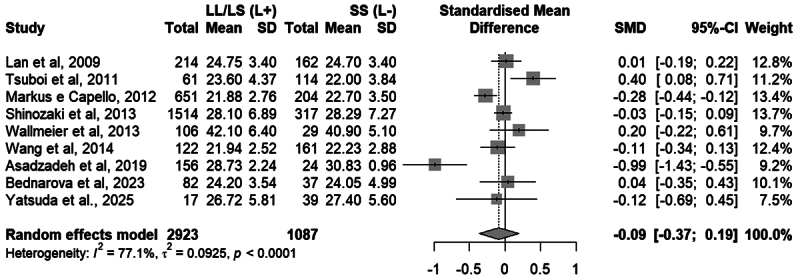
Meta-analysis of studies comparing the genotypic means of the 5-HTTLPR variant in the *SLC6A4* gene between the LL/LS and SS groups. “Total” refers to the number of individuals with the indicated genotypes. “Mean” refers to the mean BMI of the group. “SD” refers to the standard deviation. Standardized Mean Difference and 95% confidence intervals (CI) values are presented for random effect models.

Nine studies were included in the meta-analysis evaluating the association of the 5-HTTLPR variant with BMI, totaling 4,010 participants. The random-effects model indicated a standardized mean difference (SMD) of -0.0856 (95% CI: -0.3655 to 0.1943; *p* = 0.5008), with no statistical significance. Heterogeneity among studies was high and statistically significant (I² = 77.1%; *p* < 0.0001), indicating substantial variability in the observed effects. To assess the influence of outliers in the sample, the analysis was repeated after removing Asadzadeh et al. (2019). The significance of the results remained unchanged (random-effects model: SMD = -0.0105; *p* = 0.8887). Figure 7 presents the complete results.

## Discussion

### Reward-related genetic variants and their impact on BMI and obesity

Genetic variations in genes involved in the brain reward cascade can alter reward sensitivity and may play a significant role in individual susceptibility to obesity and/or increased BMI by promoting compulsive eating behaviors, long-term energy imbalance, and weight gain (Blum et al., 2000; Davis et al., 2009).

Across the selected genes, 18 variants were found to be significantly associated with BMI, overweight, or obesity. This section explores how genetic variants within these reward-related genes may contribute to increased BMI and obesity risk and how this genetic knowledge can be used to help prevent overweight and obesity in the future.

*COMT*

The *COMT* gene encodes the catechol-O-methyltransferase enzyme, which degrades catecholamines and regulates dopamine availability in the prefrontal cortex (Grossman et al., 1992; Chen et al., 2004; Dreher et al., 2009). Variants in this gene may increase dopamine catabolism and reduce synaptic function (Blum et al., 2014).

Among the 29 studies reviewed, 7 reported significant associations between *COMT* variants and BMI. The most frequently studied variant is Val158Met (rs4680), a non-synonymous SNP that results in the substitution of valine (Val) with methionine (Met) at position 158, in exon 4 of the *COMT* gene. The Met allele leads to a four-fold reduction in COMT activity, thereby increasing dopamine availability in the prefrontal cortex (Chen et al., 2004).

Given that the Val allele is associated with higher enzymatic activity and consequently lower dopamine levels, it has been hypothesized to contribute to a hypodopaminergic state and increased risk of elevated BMI. Supporting this, Val carriers were more frequent in higher BMI groups (Cribb et al., 2011), and individuals with the Val/Val genotype had higher BMI compared to those with the Val/Met genotype (Valomon et al., 2014). However, conflicting findings have also been reported, where the low activity Met allele was associated with higher BMI (Hong et al., 2003). 

Additional variants associated with BMI include His62His (rs4633) (Yang et al., 2014), a synonymous SNP (Lachman et al., 1996); Leu136Leu (rs4818), where the G allele was more frequent in individuals with obesity (Wang et al., 2007); and rs2020917, where the T allele was associated with higher BMI (Mehri et al., 2019).

*DRD2*

*DRD2* is the most extensively studied gene in relation to reward deficiency and obesity, appearing in 47 studies. It encodes the D2 dopamine receptor, a G protein-coupled receptor located on postsynaptic dopaminergic neurons, which plays a central role in reward pathways (Neville et al., 2004). *DRD2* genotypes associated with reduced dopamine signaling have been linked to altered eating behaviors, such as increased food intake frequency and preference for high-fat foods (Lek et al., 2018).

Among the variants studied, the most notable is the Taq1A variant (rs1800497), analyzed in 42 articles. Initially believed to be within *DRD2*, Taq1A is now known to reside ~10 kb downstream in the *ANKK1* gene (Savitz et al., 2013). This SNP involves a cytosine-to-thymine substitution, resulting in a glutamine-to-lysine amino acid change (Pinto et al., 2016). The A1 allele of Taq1A is associated with reduced D2 receptor expression in the striatum, with carriers exhibiting 30-40% fewer dopamine D2 receptors compared to A2 homozygotes (Noble et al., 1991; Pohjalainen et al., 1998). 

Three meta-analyses investigated the association between the A1 allele of the Taq1A variant and obesity. In the first, comparing obese individuals with those of normal weight, a significant association between the A1 allele and obesity was found in the fixed-effect model, with strong evidence according to the Venice Criteria. In the second, comparing obese individuals with non-obese controls, the fixed-effect model also showed a significant association, but the high heterogeneity among studies reduced the reliability of this result. In the third analysis, based on continuous BMI data, no significant difference was observed between carriers of the A1 allele and individuals with the A2A2 genotype, with inconsistent results across studies. Together, these findings suggest a trend toward higher prevalence of the A1 allele among individuals with obesity, supporting its role in dysfunctional eating behaviors.

In contrast, a meta-analysis by Benton and Young (2016), examining the effect of Taq1A on obesity, concluded that there was no support for the reward deficiency theory of food addiction. Other studies suggest that individuals carrying the A1 allele may respond less favorably to weight loss interventions, possibly reflecting increased impulsivity (Cameron et al., 2013; Barnard et al., 2009).

In addition to its direct influence on the dopaminergic pathway, *DRD2* may affect body weight through its interaction with *FTO*, one of the most influential genes in obesity development. An interaction between the obesity-risk polymorphism in *FTO* and Taq1A was reported, showing that those carrying both risk alleles have a substantially higher risk of obesity and diabetes (Heni et al., 2016).

Other DRD2 variants were significantly associated with obesity. The second most studied variant was −141C Ins/Del, which showed contradictory results, as both the Del allele and the Ins/Ins genotype were associated with higher BMI (Aliasghari et al., 2021; Arrue et al., 2023). Also, Taq1B (Spitz et al., 2000), where the B1 allele was more frequent in individuals with obesity, Ser311Cys (Chen et al., 2012 b ), with higher frequencies of the Cys allele in subjects with obesity compared to controls, C957T (Kvaløy et al., 2015), where the C allele appeared protective against overweight development, and rs1076560 (Morton et al., 2006), where the T allele was more frequent in individuals with normal-weight than with obesity.

*DRD4*

The DRD4 gene encodes the dopamine D4 receptor, a key modulator of dopaminergic neurotransmission (Ptáček et al., 2011). Fourteen studies have examined the association between DRD4 variants and body mass index (BMI), overweight, or obesity. 

The most extensively investigated variant is the 48 bp variable number tandem repeat (VNTR) in exon 3. Carriers of the 7-repeat (7R) allele generally present with higher BMI compared to non-carriers, while alleles with 2 or 4 repeats are often associated with lower BMI (Eisenberg et al., 2008; Levitan et al., 2010; Sikora et al., 2013; González-Giraldo et al., 2018). The 7R allele has been linked to reduced receptor responsiveness, novelty seeking and impulsivity, potentially leading to altered reward processing, preference for palatable foods, and increased susceptibility to weight gain (Eisenberg *et al*., 2008).

Another notable variant is −521C/T (rs1800955) in the promoter region, where individuals with the CC genotype showed significantly higher BMI compared to T allele carriers (Munafò et al., 2006). 

*MAO-A*

The *MAO-A* gene encodes monoamine oxidase A, a mitochondrial enzyme responsible for the oxidative deamination of neurotransmitters such as dopamine and serotonin (Guglielmi et al., 2022). Among the seven studies that investigated *MAO-A* variants in relation to BMI, six focused on the 30 bp VNTR. This variant involves 3, 3.5, 4, or 5 repeats of a 30-base-pair sequence, with functional consequences on gene transcription. Alleles with 3.5 or 4 repeats are considered high-activity variants, transcribed 2-10 times more efficiently than the low-activity alleles (3 or 5 repeats), suggesting an “optimum length” of promoter activity (Sabol et al., 1998).

Two studies reported significant associations, showing that carriers of low-activity alleles had higher BMI, possibly due to reduced monoamine degradation and consequent alterations in reward signaling. As *MAO-A* is located on the X chromosome, these effects may be more pronounced in men, who carry only one copy of the gene, increasing their susceptibility to the influence of functional variants on body weight regulation (Ducci et al., 2006; Need et al., 2006).

*SLC6A3*

The *SLC6A3* gene, also known as *DAT1*, encodes the dopamine transporter protein, which regulates synaptic dopamine by reuptaking extracellular dopamine into presynaptic terminals. This function directly influences the duration and intensity of dopaminergic signaling, especially in brain regions involved in reward and motivation (Dreher et al., 2009).

The most studied variant is the 40 bp VNTR (rs28363170) located in the 3’ untranslated region (3’UTR) of exon 15 (Vandenbergh et al., 1992). This VNTR exists primarily in 9-repeat (9R) and 10-repeat (10R) alleles. Functional studies suggest that the 10R allele is associated with higher expression of the transporter, leading to enhanced dopamine reuptake and lower extracellular dopamine levels, which may contribute to a hypodopaminergic state (VanNess et al., 2005). Consistent with this mechanism, the 10R/10R genotype has been repeatedly associated with higher BMI and obesity risk (Epstein et al., 2002; González-Giraldo et al., 2018), whereas the 9R allele has been linked to lower BMI and reduced obesity risk (Azzato et al., 2009). However, conflicting evidence exists, as some studies reported higher BMI in 9R homozygotes (Bieliński et al., 2017), highlighting possible population-specific effects.

Additional variants of *SLC6A3* have also been implicated in body weight regulation. The 1215A>G variant (Ex9-55) was more frequent in underweight individuals carrying the G allele (Azzato et al., 2009), suggesting a protective role against weight gain. The G2319A variant (rs27072) was associated with higher BMI in carriers of the G allele, both heterozygous and homozygous, compared to AA homozygotes (Pavlova et al., 2019).

*SLC6A4*

The *SLC6A4* gene encodes the serotonin transporter, which regulates serotonin reuptake from the synaptic cleft into presynaptic neurons. Serotonin plays a central role in mood, appetite regulation, and satiety, with its signaling being critically modulated by this transporter (Heils et al., 1996; Grimm and Steinle, 2011).

The most widely studied variant in *SLC6A4* is the 5-HTTLPR, a functional insertion/deletion located in the promoter region, in the form of a long (L) or short (S) allele. The L allele is associated with higher transcriptional activity, leading to increased expression of the serotonin transporter and greater serotonin reuptake, thereby reducing serotonin levels in the synaptic cleft (Heils et al., 1996). On the other hand, the S allele is associated with increased vulnerability to anxiety and depression. These disorders, in turn, show a significantly higher prevalence among individuals with obesity, suggesting that the S allele may indirectly contribute to weight gain risk through mental health-related mechanisms (Sookoian et al., 2008).

In studies examining the relationship between 5-HTTLPR and BMI, findings have been inconsistent. Some studies reported a higher prevalence of obesity among individuals with the S allele (Sookoian et al., 2008; Asadzadeh et al., 2019), while other found that L allele carriers (LL or LS genotypes) had significantly higher BMI than SS individuals (Peralta-Leal et al., 2012; Borkowska et al., 2015; Galaviz-Hernández et al., 2020), particularly in subgroups such as individuals with high neuroticism or low physical activity (Markus and Capello, 2012; Dias et al., 2016). 

Two meta-analyses were conducted to evaluate the effect of the 5-HTTLPR variant on BMI and obesity. In the categorical analysis, no significant association was found between the long (L) allele and the risk of overweight/obesity, with moderate heterogeneity among studies. In the continuous analysis, no significant difference in BMI was observed between individuals with and without the L allele, with small, inconsistent effects and wide confidence intervals. Analyses considering the S allele as the risk variant also showed no significant associations.

### Synthesized results

Across the 18 variants significantly associated with the six genes of interest, five variants were consistently associated in the literature, as shown in Table 2.

**Table 2 -:** Summary of qualitative findings.

Gene	Variant	Risk allele
*COMT*	Val158Met (rs4680)	Val
*DRD2*	Taq1A (rs1800497)	A1
*DRD4*	48 bp VNTR	7R
*SLCC6A3*	40 bp VNTR	10R
*SLCC6A4*	5-HTTLPR	L

Val: Valine; 7R: 7 repetitions; 10R: 10 repetitions; L: Long allele.

**Figure 8 -:**
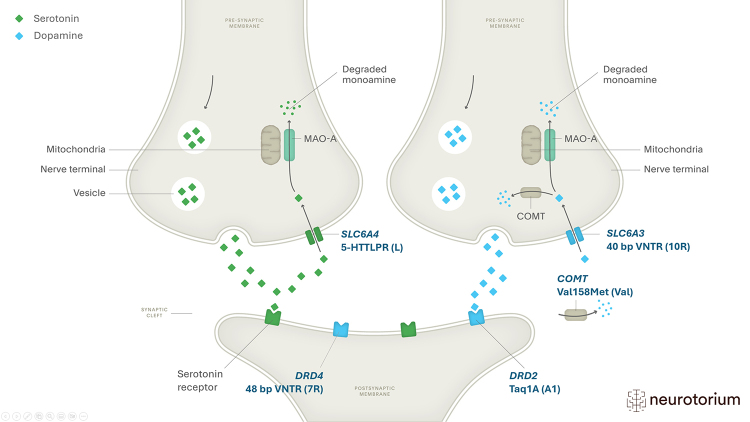
Schematic representation of the main variants acting in the synapse. Prepared by the authors (2025). Adapted from: https://neurotorium.org/image/serotonin-and-noradrenaline-synaptic-activity-2/

The selected variants influence the production of dopamine and serotonin receptors, transporters, and degrading enzymes. Figure 8 illustrates the role of these variants during the synapse and highlights the risk variants identified in the literature.

A total of five meta-analyses were conducted to evaluate the effect of the Taq1A A1 allele and the 5-HTTLPR Long allele on increased BMI and the predisposition to overweight and obesity. Table 3 summarizes the findings of these meta-analyses.

**Table 3 -:** Summary of meta-analyses results.

Meta-analysis	Result
Frequency of the A1 allele (Taq1A): obesity group (BMI ≥ 30) vs normal-weight group (BMI < 25).	Fixed: OR = 1.22; 95% CI = 1.05-1.43; ****p* = 0.0096*** Random: OR = 1.23; 95% CI = 0.99-1.51; *p* = 0.0562
Frequency of the A1 allele (Taq1A): obesity group (BMI ≥ 30) vs overweight/normal weight (BMI < 30).	Fixed: OR = 1.31; 95% CI = 1.16 - 1.48; ****p* < 0.0001*** Random: OR = 1.50; 95% CI = 0.87-2.57; *p* = 0.1138
Genotypic means (Taq1A): A1+ (A1A1/A1A2) vs A1- (A2A2)	Random: SMD = 0.02; 95% CI = -0.14 - 0.19; *p* = 0.7561:
Frequency of the L allele: Overweight/Obesity (BMI ≥ 25) vs normal-weight group (BMI < 25).	Fixed: OR = 1.01; 95% CI = 0.92 - 1.11; *p* = 0.7864 Random: OR = 1.05; 95% CI = 0.84 - 1.31; *p* = 0.5840
Genotypic means: L+ (LL/LS) vs L- (SS)	Random: SMD = -0.09; 95% CI = -0.37 - 0.19; *p* = 0.5008

OR = Odds ratio; SMD = Standardized mean deviation; CI = Confidence interval.

### Limitations and risk of bias

A comprehensive and systematic search was conducted across multiple databases. However, no studies involving Brazilian or African populations, which are an important source of genetic ancestry in Brazil (Nunes et al., 2025), met the predefined inclusion criteria. This absence of eligible studies does not necessarily indicate a lack of association but rather highlights an important gap in the literature and reinforces the need for future research focusing on these underrepresented groups. 

The included studies exhibited recurring limitations. Ancestry was rarely reported, despite evidence that BMI tends to overestimate body fat in African Americans (Liu et al., 2024). BMI was frequently categorized using nonstandard criteria, and data presentation was inconsistent, varying between means, frequencies, genotypes, or alleles. This limited comparability and the feasibility of conducting meta-analysis. Of the 117 studies, only 31 could be included in the meta-analyses, primarily due to missing allele or genotype frequencies, atypical BMI categorizations, incomplete or non-standardized measures, unconventional genotype groupings, or results presented exclusively in graphical form without raw data.

## Conclusion

This study advances the understanding of the biological mechanisms linking the reward cascade to eating behavior and obesity risk. By integrating the available evidence, we identified consistent associations between specific genetic variants and predisposition to weight gain, providing a basis for more precise prevention and treatment strategies. In the future, these variants may contribute to a genetic risk panel in which the accumulation of risk alleles indicates greater susceptibility to weight gain through neurobehavioral pathways associated with reward deficiency. Such information could enable early preventive interventions, reducing reliance on pharmacological approaches and supporting personalized obesity management.

Future studies should adopt the BMI classification recommended by the WHO and account for its particularities across different populations. Data presentation should, whenever possible, include both genotype frequencies and means to enhance comparability across studies and facilitate meta-analyses. Additional studies are needed in Brazil and Africa, and future research should also incorporate ancestry as a key variable in sample characterization.

## Supplementary material

The following online material is available for this article:

Table S1 - Complete search strategies.

Table S2 - PRISMA checklist.

Table S3 - Variants studied without association.

Table S4 - List of articles included in the systematic review.

Table S5 - Risk of bias for each included study.

## Data Availability

The data from this study have not been published and are not under consideration elsewhere.
