# Supplementary material for: Influence of reward-related genetic variants on BMI and predisposition to obesity: Systematic review and meta-analysis
Source: Genet Mol Biol. 2026 May 22;49(Suppl 1):e20250216. doi: 10.1590/1678-4685-GMB-2025-0216 (PMC13196784; doi:10.1590/1678-4685-GMB-2025-0216)
Supplement: Table S1 - [file 1415-4757-GMB-49-s1-e20250216-s1.pdf]

Genetics and Molecular Biology
https://doi.org/10.1590/1678-4685-GMB-2025-0216

Supplementary Material to "Influence of reward-related genetic variants on BMI and predisposition to obesity: Systematic review and meta-analysis"

Table S1 – Complete search strategies, divided by database and number of articles retrieved.

Database	Search strategy	Results
**PubMed**	("COMT"[Title/Abstract] OR "DRD2"[Title/Abstract] OR "ANKK1"[Title/Abstract] OR "DRD4"[Title/Abstract] OR "MAOA"[Title/Abstract] OR "MAO-A"[Title/Abstract] OR "SLC6A3"[Title/Abstract] OR "DAT1"[Title/Abstract] OR "SLC6A4"[Title/Abstract] OR "SERT" OR "5-HTTLPR"[Title/Abstract]) AND ("Body Mass Index"[Title/Abstract] OR "BMI"[Title/Abstract] OR "Overweight"[Title/Abstract] OR "Obesity"[Title/Abstract] OR "Obese"[Title/Abstract])	432
**Scopus**	TITLE-ABS-KEY ("COMT" OR "DRD2" OR "ANKK1" OR "DRD4" OR "MAOA" OR "MAO-A" OR "SLC6A3" OR "DAT1" OR "SLC6A4" OR "SERT" OR "5-HTT" OR "5-HTTLPR") AND TITLE-ABS-KEY ("Body Mass Index" OR "BMI" OR "Overweight" OR "Obesity" OR "Obese") AND (LIMIT-TO (DOCTYPE , "ar")) AND (LIMIT-TO (LANGUAGE , "English") OR LIMIT-TO (LANGUAGE , "Spanish") OR LIMIT-TO (LANGUAGE , "Portuguese")) AND (LIMIT-TO (PUBSTAGE , "final")) AND (LIMIT-TO (SRCTYPE , "j"))	625
**Embase**	('comt':ti,ab,kw OR 'drd2':ti,ab,kw OR 'ankk1':ti,ab,kw OR 'drd4':ti,ab,kw OR 'maoa':ti,ab,kw OR 'mao-a':ti,ab,kw OR 'slc6a3':ti,ab,kw OR 'dat1':ti,ab,kw OR 'slc6a4':ti,ab,kw OR 'sert':ti,ab,kw OR '5-htt':ti,ab,kw OR '5-httlpr':ti,ab,kw) AND ('body mass index':ti,ab,kw OR bmi:ti,ab,kw OR overweight:ti,ab,kw OR obesity:ti,ab,kw OR obese:ti,ab,kw) AND ('article'/it OR 'clinical trial'/it) AND [embase]/lim NOT [medline]/lim	72

Database	Search strategy	Results
**LILACS**	("COMT" OR "DRD2" OR "ANKK1" OR "DRD4" OR "MAOA" OR "MAO-A" OR "SLC6A3" OR "DAT1" OR "SLC6A4" OR "SERT" OR "5-HTT" OR "5-HTTLPR")) AND (("Body Mass Index" OR "BMI" OR "IMC" OR "Índice de massa corporal" OR "Índice de masa corporal" OR "Overweight" OR "Sobrepeso" OR "Obesity" OR "Obesidade" OR "Obese" OR "Obeso")) AND db:("PubMed-not-MEDLINE") AND instance:"lilacsplus"	6
**Web of Science**	TS=("COMT" OR "DRD2" OR "ANKK1" OR "DRD4" OR "MAOA" OR "MAO-A" OR "SLC6A3" OR "DAT1" OR "SLC6A4" OR "SERT" OR "5-HTT" OR "5-HTTLPR") AND TS=("Body Mass Index" OR "BMI" OR "Overweight" OR "Obesity" OR "Obese")	633
**Scholar Google**	("COMT" OR "DRD2" OR "ANKK1" OR "DRD4" OR "MAOA" OR "MAO-A" OR "SLC6A3" OR "DAT1" OR "SLC6A4" OR "SERT" OR "5-HTT" OR "5-HTTLPR") AND ("Body Mass Index" OR "BMI" OR "Overweight" OR "Obesity" OR "Obese")	100
**Total**		**1,868**

Searches conducted in July 2025.
