# Supplementary material for: Influence of reward-related genetic variants on BMI and predisposition to obesity: Systematic review and meta-analysis
Source: Genet Mol Biol. 2026 May 22;49(Suppl 1):e20250216. doi: 10.1590/1678-4685-GMB-2025-0216 (PMC13196784; doi:10.1590/1678-4685-GMB-2025-0216)
Supplement: Table S3 - [file 1415-4757-GMB-49-s1-e20250216-s3.pdf]

Genetics and Molecular Biology
https://doi.org/10.1590/1678-4685-GMB-2025-0216

Supplementary Material to "Influence of reward-related genetic variants on BMI and predisposition to obesity: Systematic review and meta-analysis"

Table S3 – Variants studied without association.

Gene	Variants	Studies that did not show association
COMT	His62His (rs4633)	Wang *et al.*, 2007<br>Hill *et al.*, 2012
	Leu136Leu (rs4818)	Hill *et al.*, 2012
	rs2075507	Mehri *et al.*, 2019
	rs6269	Hill *et al.*, 2012
	Val158Met (rs4680)	Lavigne *et al.*, 1997<br>Thompson *et al.*, 1998<br>Millikan *et al.*, 1998<br>Mitrunen *et al.*, 2001<br>Yim *et al.*, 2001<br>Kocabaş *et al.*, 2002<br>Tworoger *et al.*, 2004<br>Kocabaş *et al.*, 2005<br>Need *et al.*, 2006<br>Gorai *et al.*, 2007

Gene	Variants	Studies that did not show association
		Wang *et al.*, 2007
		Annerbrink *et al.*, 2008
		Justenhoven *et al.*, 2008
		Kring *et al.*, 2009
		Lloret Linares *et al.*, 2011
		Witte *et al.*, 2010
		Hill *et al.*, 2012
		Thaler *et al.*, 2012
		Hursel *et al.*, 2014
		Yokum *et al.*, 2015
		Jawinski *et al.*, 2016
		Arrue *et al.*, 2023
		Losada-Casallas *et al.*, 2024
DRD2	Taq1A (rs1800497)	Noble *et al.*, 1994<br>Blum *et al.*, 1996<br>Spitz *et al.*, 2000<br>Thomas *et al.*, 2000<br>Southon *et al.*, 2003<br>Zhang *et al.*, 2003<br>Fang *et al.*, 2005<br>Epstein *et al.*, 2004<br>Epstein *et al.*, 2007

Gene	Variants	Studies that did not show association
		Davis *et al.*, 2008
		Barnard *et al.*, 2009
		Davis *et al.*, 2009
		Epstein *et al.*, 2010
		Ariza *et al.*, 2012
		Cameron *et al.*, 2013
		Nisoli *et al.*, 2007
		Roth *et al.*, 2013
		Athanasoulia *et al.*, 2014
		Yokum *et al.*, 2015
		Yeh *et al.*, 2016
		Lek *et al.*, 2018
		Rivera-Iñiguez *et al.*, 2019
		Frank *et al.*, 2018
		Palacios *et al.*, 2018
		Ramos-Lopez *et al.*, 2019
		Lim *et al.*, 2020
		Gassó *et al.*, 2020
		Matsunaga *et al.*, 2021
		Aliasghari *et al.*, 2021a
		Beyer *et al.*, 2021
		Obregón *et al.*, 2022

Gene	Variants	Studies that did not show association
		Daza-Hernández *et al.*, 2023
	Taq1B (rs1079597)	Lek *et al.*, 2018<br>Lim *et al.*, 2020
	Ser311Cys (rs1801028)	Southon *et al.*, 2003
	C957T (rs6277)	Morton *et al.*, 2006<br>Davis *et al.*, 2008<br>Davis *et al.*, 2009
	−241 A/G (rs1799978)	Morton *et al.*, 2006<br>Davis *et al.*, 2008
	−141C Ins/Del (rs1799732)	Davis *et al.*, 2008<br>Davis *et al.*, 2009<br>Frank *et al.*, 2018<br>Aliasghari *et al.*, 2021b<br>Hidalgo Vira *et al.*, 2023<br>Daza-Hernández *et al.*, 2023
	Taq1D (rs1800498)	Davis *et al.*, 2008<br>Lek *et al.*, 2018<br>Lim *et al.*, 2020
	(rs4648317)	Davis *et al.*, 2008<br>Gassó *et al.*, 2020
	(rs4938013)	Palacios *et al.*, 2018
	(rs1124491)	Gassó *et al.*, 2020

Gene	Variants	Studies that did not show association
	(rs2234689)	Gassó *et al.*, 2020
	(rs2734833)	Gassó *et al.*, 2020
	(rs17529477)	Gassó *et al.*, 2020
	(rs7131056)	Gassó *et al.*, 2020
	(rs10891556)	Gassó *et al.*, 2020
DRD4	48 bp VNTR	Poston 2nd *et al.*, 1998<br>Ariza *et al.*, 2012<br>Thaler *et al.*, 2012<br>Roth *et al.*, 2013<br>Yokum *et al.*, 2015<br>Uzun *et al.*, 2015<br>Jawinski *et al.*, 2016<br>Arrue *et al.*, 2023<br>Losada-Casallas *et al.*, 2024
MAO-A	EcoRV	Camarena *et al.*, 2004
	30 bp VNTR	Camarena *et al.*, 2004<br>Need *et al.*, 2006<br>Brummet *et al.*, 2008<br>Wallmeier *et al.*, 2013<br>Dias *et al.*, 2016
	rs1801291	Gallicchio *et al.*, 2009
SLC6A3	40 bp VNTR (rs28363170)	Epstein *et al.*, 2004

Gene	Variants	Studies that did not show association
		Need *et al.*, 2006<br>Epstein *et al.*, 2007<br>Thaler *et al.*, 2012<br>Valomon *et al.*, 2014<br>Yokum *et al.*, 2015<br>Uzun *et al.*, 2015<br>Arrue *et al.*, 2023<br>Losada-Casallas *et al.*, 2024
SLC6A4	5-HTTLPR	Mergen *et al.*, 2007<br>Lan *et al.*, 2009<br>Lee *et al.*, 2009<br>Iordanidou *et al.*, 2010<br>Bah *et al.*, 2010<br>Suriyaprom *et al.*, 2012<br>Markus and Capello, 2012<br>Shinozaki *et al.*, 2012<br>Wallmeier *et al.*, 2013<br>Shinozaki *et al.*, 2013<br>Capello and Markus, 2014a<br>Capello and Markus, 2014b<br>Wang *et al.*, 2014<br>Markus *et al.*, 2015

Gene	Variants	Studies that did not show association
		Hameed *et al.*, 2015
		Hinderberger *et al.*, 2016
		Schepers *et al.*, 2017
		Bednarova *et al.*, 2023
	5-HTTVNTR	Lee *et al.*, 2009
		Uzun *et al.*, 2015
	rs16965628	Paderina *et al.*, 2021

Variants that were not significantly associated with BMI or obesity.

References

Aliasghari F, Mahdavi R, Barati M, Nazm SA, Yasari S, Bonyadi M and Jabbari M (2021a) Genotypes of ANKK1 and DRD2 genes and risk of metabolic syndrome and its components: A cross-sectional study on Iranian women. Obes Res Clin Pract 15:449–454.

Aliasghari F, Pirdehghan A, Aghamohammadzadeh N, Rashtchizadeh N, Azarfam P and Yaghmaei P (2021b) Associations of the ANKK1 and DRD2 gene variants with overweight, obesity and hedonic hunger among women from the Northwest of Iran. Eat Weight Disord 26:305–312.

Annerbrink K, Westberg L, Nilsson S, Rosmond R, Holm G and Eriksson E (2008) Catechol O-methyltransferase val158-met variant is associated with abdominal obesity and blood pressure in men. Metabolism 57:708–711.

Ariza M, Garolera M, Jurado MA, Garcia-Garcia I, Hernan I, Sánchez-Garre C, Vernet-Vernet M, Sender-Palacios MJ, Marques-Iturria I, Pueyo R *et al.* (2012) Dopamine genes (DRD2/ANKK1-TaqA1 and DRD4-7R) and executive function: Their interaction with obesity. PLoS One 7:e41482.

Arrue A, Olivas O, Erkoreka L, Alvarez FJ, Arnaiz A, Varela N, Bilbao A, Rodríguez JJ, Moreno-Calle MT and Gordo E (2023) Multilocus genetic profile reflecting low dopaminergic signaling is directly associated with obesity and cardiometabolic disorders due to antipsychotic treatment. Pharmaceutics 15:2134.

Athanasoulia AP, Sievers C, Uhr M, Ising M, Stalla GK and Schneider HJ (2014) The effect of the ANKK1/DRD2 Taq1A variant on weight changes of dopaminergic treatment in prolactinomas. Pituitary 17:240–245.

Bah J, Westberg L, Baghaei F, Henningsson S, Rosmond R, Melke J, Holm G and Eriksson E (2010) Further exploration of the possible influence of variants in HTR2C and 5HTT on body weight. Metabolism 59:1156–1163.

Barnard ND, Noble EP, Ritchie T, Cohen J, Jenkins DJ, Turner-McGrievy G, Gloede L, Green AA and Ferdowsian H (2009) D2 dopamine receptor Taq1A variant, body weight, and dietary intake in type 2 diabetes. Nutrition 25:58–65.

Bednarova A, Habalova V, Iannaccone SF, Tkac I, Jarcuskova D, Krivosova M, Marcatili M and Hlavacova N (2023) Association of HTTLPR, BDNF, and FTO genetic variants with completed suicide in Slovakia. J Pers Med 13:501.

Beyer F, Zhang R, Scholz M, Wirkner K, Loeffler M, Stumvoll M, Villringer A and Witte AV (2021) Higher BMI, but not obesity-related genetic variants, correlates with lower structural connectivity of the reward network in a population-based study. Int J Obes (Lond) 45:491–501.

Blum K, Braverman ER, Wood RC, Gill J, Li C, Chen TJ, Taub M, Montgomery AR, Sheridan PJ and Cull JG (1996) Increased prevalence of the TaqI A1 allele of the dopamine receptor gene (DRD2) in obesity with comorbid substance use disorder: a preliminary report. Pharmacogenetics 6:297–305.

Brummett BH, Boyle SH, Siegler IC, Zuchner S, Ashley-Koch A and Williams RB (2008) Lipid levels are associated with a regulatory variant of the monoamine oxidase-A gene promoter (MAOA-uVNTR). Med Sci Monit 14:CR57–CR61.

Camarena B, Santiago H, Aguilar A, Ruvinskis E, González-Barranco J and Nicolini H (2004) Family-based association study between the monoamine oxidase A gene and obesity: Implications for psychopharmacogenetic studies. Neuropsychobiology 49:126–129.

Cameron JD, Riou MÈ, Tesson F, Goldfield GS, Rabasa-Lhoret R, Brochu M and Doucet É (2013) The TaqIA RFLP is associated with attenuated intervention-induced body weight loss and increased carbohydrate intake in post-menopausal obese women. Appetite 60:111–116.

Capello AE and Markus CR (2014a) Differential influence of the 5-HTTLPR genotype, neuroticism and real-life acute stress exposure on appetite and energy intake. Appetite 77:83–93.

Capello AE and Markus CR (2014b) Effect of sub chronic tryptophan supplementation on stress-induced cortisol and appetite in subjects differing in 5-HTTLPR genotype and trait neuroticism. Psychoneuroendocrinology 45:96–107.

Davis C, Levitan RD, Kaplan AS, Carter J, Reid C, Curtis C, Patte K, Hwang R and Kennedy JL (2008) Reward sensitivity and the D2 dopamine receptor gene: A case-control study of binge eating disorder. Prog Neuropsychopharmacol Biol Psychiatry 32:620–628.

Davis CA, Levitan RD, Reid C, Carter JC, Kaplan AS, Patte KA, King N, Curtis C and Kennedy JL (2009) Dopamine for "wanting" and opioids for "liking": A comparison of obese adults with and without binge eating. Obesity (Silver Spring) 17:1220–1225.

Daza-Hernández S, Pérez-Luque E, Martínez-Cordero C, Figueroa-Vega N, Cardona-Alvarado MI and Muñoz-Montes N (2023) Analysis of factors associated with outcomes of bariatric surgery: rs1800497 ANKK1, rs1799732 DRD2 genetic variants, eating behavior, hedonic hunger, and depressive symptoms. J Gastrointest Surg 27:1778-1784.

Dias H, Muc M, Padez C and Manco L (2016) Association of variants in 5-HTT (SLC6A4) and MAOA genes with measures of obesity in young adults of Portuguese origin. Arch Physiol Biochem 122:8–13.

Epstein LH, Wright SM, Paluch RA, Leddy JJ, Hawk LW Jr, Jaroni JL, Saad FG, Crystal-Mansour S, Shields PG and Lerman C (2004) Relation between food reinforcement and dopamine genotypes and its effect on food intake in smokers. Am J Clin Nutr 80:82–88.

Epstein LH, Temple JL, Neaderhiser BJ, Salis RJ, Erbe RW and Leddy JJ (2007) Food reinforcement, the dopamine D2 receptor genotype, and energy intake in obese and nonobese humans. Behav Neurosci 121:877–886.

Epstein LH, Dearing KK and Erbe RW (2010) Parent-child concordance of Taq1 A1 allele predicts similarity of parent-child weight loss in behavioral family-based treatment programs. Appetite 55:363–366.

Fang YJ, Thomas GN, Xu ZL, Fang JQ and Critchley JA and Tomlinson B (2005) An affected pedigree member analysis of linkage between the dopamine D2 receptor gene TaqI variant and obesity and hypertension. Int J Cardiol 102:111–116.

Frank GKW, Shott ME, DeGuzman MC and Smolen A (2018) Dopamine D2 -141C Ins/Del and Taq1A variants, body mass index, and prediction error brain response. Transl Psychiatry 8:102.

Gallicchio L, Chang HH, Christo DK, Thuita L, Huang HY, Strickland P, Ruczinski I, Clipp S and Helzlsouer KJ (2009) Single nucleotide variants in obesity-related genes and all-cause and cause-specific mortality: A prospective cohort study. BMC Med Genet 10:103.

Gassó P, Arnaiz JA, Mas S, Lafuente A, Bioque M, Cuesta MJ, Díaz-Caneja CM, García C, Lobo A, González-Pinto A *et al.* (2020) Association study of candidate genes with obesity and metabolic traits in antipsychotic-treated patients with first-episode psychosis over a 2-year period. J Psychopharmacol 34:514–523.

Gorai I, Inada M, Morinaga H, Uchiyama Y, Yamauchi H, Hirahara F and Chaki O (2007) CYP17 and COMT gene variants can influence bone directly, or indirectly through their effects on endogenous sex steroids, in postmenopausal Japanese women. Bone 40:28–36.

Hameed A, Ajmal M, Nasir M and Ismail M (2015) Genetic association analysis of serotonin transporter variant (5-HTTLPR) with type 2 diabetes patients of Pakistani population. Diabetes Res Clin Pract 108:67–71.

Hidalgo Vira N, Oyarce K, Valladares Vega M, Goldfield GS, Guzmán-Gutiérrez E and Obregón AM (2023) No association of the dopamine D2 receptor genetic bilocus score (rs1800497/rs1799732) on food addiction and food reinforcement in Chilean adults. Front Behav Neurosci 17:1067384.

Hill LD, Ewens KG, Maher BS, York TP and Legro RS, Dunaif A and Strauss 3rd JF (2012) Catechol-O-methyltransferase (COMT) single nucleotide variants and haplotypes are not major risk factors for polycystic ovary syndrome. Mol Cell Endocrinol 350:72–77.

Hinderberger P, Rullmann M, Drabe M, Luthardt J, Becker GA, Blüher M, Regenthal R, Sabri O and Hesse S (2016) The effect of serum BDNF levels on central serotonin transporter availability in obese versus non-obese adults: A [(11)C]DASB positron emission tomography study. Neuropharmacology 110:530–536.

Hursel R, Janssens PL, Bouwman FG, Mariman EC and Westerterp-Plantenga MS (2014) The role of catechol-O-methyl transferase Val(108/158)Met variant (rs4680) in the effect of green tea on resting energy expenditure and fat oxidation: a pilot study. PLoS One 9:e106220.

Iordanidou M, Tavridou A, Petridis I, Arvanitidis KI, Christakidis D, Vargemezis V and Manolopoulos VG (2010) The serotonin transporter promoter variant (5-HTTLPR) is associated with type 2 diabetes. Clin Chim Acta 411:167–171.

Jawinski P, Tegelkamp S, Sander C, Häntzsch M, Huang J, Mauche N, Scholz M, Spada J, Ulke C, Burkhardt R *et al.* (2016) Time to wake up: No impact of COMT Val158Met gene variation on circadian preferences, arousal regulation and sleep. Chronobiol Int 33:893–905.

Justenhoven C, Hamann U, Schubert F, Zapatka M, Pierl CB, Rabstein S, Selinski S, Mueller T, Ickstadt K, Gilbert M *et al.* (2008) Breast cancer: A candidate gene approach across the estrogen metabolic pathway. Breast Cancer Res Treat 137:49.

Kocabaş NA, Sardaş S, Cholerton S, Daly AK and Karakaya AE (2002) Cytochrome P450 CYP1B1 and catechol O-methyltransferase (COMT) genetic variants and breast cancer susceptibility in a Turkish population. Arch Toxicol 76:643–649.

Kocabaş NA, Sardaş S and Karakaya AE (2005) Variants related to estrogen and xenobiotic metabolism in healthy Turkish women. Arch Med Res 36:19–23.

Kring SI, Werge T, Holst C, Toubro S, Astrup A, Hansen T, Pedersen O and Sørensen TI (2009) Variants of serotonin receptor 2A and 2C genes and COMT in relation to obesity and type 2 diabetes. PLoS One 4:e6696.

Lan MY, Chang YY, Chen WH, Kao YF, Lin HS and Liu JS (2009) Serotonin transporter gene promoter variant is associated with body mass index and obesity in non-elderly stroke patients. J Endocrinol Invest 32:119–122.

Lavigne JA, Helzlsouer KJ, Huang HY, Strickland PT, Bell DA, Selmin O, Watson MA, Hoffman S, Comstock GW and Yager JD (1997) An association between the allele coding for a low activity variant of catechol-O-methyltransferase and the risk for breast cancer. Cancer Res 57:5493–5497.

Lee HY, Kim DJ, Lee HJ, Choi JE and Kim YK (2009) No association of serotonin transporter variant (5-HTTVNTR and 5-HTTLPR) with characteristics and treatment response to atypical antipsychotic agents in schizophrenic patients. Prog Neuropsychopharmacol Biol Psychiatry 33:276–280.

Lek FY, Ong HH and Say YH (2018) Association of dopamine receptor D2 gene (DRD2) Taq1 variants with eating behaviors and obesity among Chinese and Indian Malaysian university students. Asia Pac J Clin Nutr 27:707–717.

Lim ZM, Chie QT and Teh LK (2020) Influence of dopamine receptor gene on eating behaviour and obesity in Malaysia. Meta Gene 25:100736.

Lloret Linares C, Hajj A, Poitou C, Simoneau G, Clement K, Laplanche JL, Lépine JP, Bergmann JF, Mouly S and Peoc'h K (2011) Pilot study examining the frequency of several gene variants involved in morphine pharmacodynamics and pharmacokinetics in a morbidly obese population. Obes Surg 21:1257–1264.

Losada-Casallas K, Cepeda-Leal I, Ruiz N, Muñoz-Ospina B and Ortega-Avila G (2024) Body index mass not associated with DRD4, DAT1, BDNF, and COMT gene variants in young adults without depression or anxiety disorders. Genet Mol Res 23:gmr19161.

Markus CR and Capello AE (2012) Contribution of the 5-HTTLPR gene by neuroticism on weight gain in male and female participants. Psychiatr Genet 22:279–285.

Matsunaga M, Ohtsubo Y, Masuda T, Noguchi Y, Yamasue H and Ishii K (2021) A genetic variation in the Y chromosome among modern Japanese males related to several physiological and psychological characteristics. Front Behav Neurosci 15:774879.

Mehri F, Tahmasebi Fard Z and Ghoraeian P (2019) The investigation of functional genetic variation in COMT gene promoter (rs2020917 & rs2075507) in Iranian patients with breast cancer. Int J Cancer Manag 12:e92008.

Mergen H, Karaaslan C, Mergen M, Deniz Ozsoy E and Ozata M (2007) LEPR, ADBR3, IRS-1 and 5-HTT genes variants do not associate with obesity. Endocr J 54:89-94.

Millikan RC, Pittman GS, Tse CK, Duell E, Newman B, Savitz D, Moorman PG, Boissy RJ and Bell DA (1998) Catechol-O-methyltransferase and breast cancer risk. Carcinogenesis 19:1943–1947.

Mitrunen K, Jourenkova N, Kataja V, Eskelinen M, Kosma VM, Benhamou S, Kang D, Vainio H, Uusitupa M and Hirvonen A (2001) Polymorphic catechol-O-methyltransferase gene and breast cancer risk. Cancer Epidemiol Biomarkers Prev 10:635–640.

Morton LM, Wang SS, Bergen AW, Chatterjee N, Kvale P, Welch R, Yeager M, Hayes RB, Chanock SJ and Caporaso NE (2006) DRD2 genetic variation in relation to smoking and obesity in the Prostate, Lung, Colorectal, and Ovarian Cancer Screening Trial. Pharmacogenet Genomics 16:901–910.

Need AC, Ahmadi KR, Spector TD and Goldstein DB (2006) Obesity is associated with genetic variants that alter dopamine availability. Ann Hum Genet 70:293–303.

Nisoli E, Brunani A, Borgomainerio E, Tonello C, Dioni L, Briscini L, Redaelli G, Molinari E, Cavagnini F and Carruba MO (2007) D2 dopamine receptor (DRD2) gene Taq1A variant and the eating-related psychological traits in eating disorders (anorexia nervosa and bulimia) and obesity. Eat Weight Disord 12:91–96.

Noble EP, Noble RE, Ritchie T, Syndulko K, Bohlman MC, Noble LA, Zhang Y, Sparkes RS and Grandy DK (1994) D2 dopamine receptor gene and obesity. Int J Eat Disord 15:205–217.

Obregón AM, Oyarce K, García-Robles MA, Valladares M, Pettinelli P and Goldfield GS (2022) Association of the dopamine D2 receptor rs1800497 variant with food addiction, food reinforcement, and eating behavior in Chilean adults. Eat Weight Disord 27:215–224.

Paderina DZ, Boiko AS, Pozhidaev IV, Bocharova AV, Mednova IA, Fedorenko OY, Kornetova EG, Loonen AJM, Semke AV, Bokhan NA *et al.* (2021) Genetic variants of 5-HT receptors and antipsychotic-induced metabolic dysfunction in patients with schizophrenia. J Pers Med 11:181.

Palacios A, Canto P, Tejeda ME, Stephano S, Luján H, García-García E, Rojano-Mejía D and Méndez JP (2018) Complete sequence of the ANKK1 gene in Mexican-Mestizo individuals with obesity, with or without binge eating disorder. Eur Psychiatry 54:59–64.

Poston 2nd WS, Ericsson M, Linder J, Haddock CK, Hanis CL, Nilsson T, Aström M and Foreyt JP (1998) D4 dopamine receptor gene exon III variant and obesity risk. Eat Weight Disord 3:71–77.

Ramos-Lopez O, Mejia-Godoy R, Frías-Delgadillo KJ, Torres-Valadez R, Flores-García A, Sánchez-Enríquez S, Aguiar-García P, Martínez-López E and Zepeda-Carrillo EA (2019) Interactions between DRD2/ANKK1 TaqIA variant and dietary factors influence plasma triglyceride concentrations in diabetic patients from Western Mexico: A cross-sectional study. Nutrients 11:2863.

Rivera-Iñiguez I, Panduro A, Ramos-Lopez O, Villaseñor-Bayardo SJ and Roman S (2019) DRD2/ANKK1 TaqI A1 variant associates with overconsumption of unhealthy foods and biochemical abnormalities in a Mexican population. Eat Weight Disord 24:835–844.

Markus CR, Jonkman LM, Capello A, Leinders S and Hüsch F (2015) Sucrose preload reduces snacking after mild mental stress in healthy participants as a function of 5-hydroxytryptamine transporter gene promoter variant. Stress 18:149–159.

Roth CL, Hinney A, Schur EA, Elfers CT and Reinehr T (2013) Association analyses for dopamine receptor gene variants and weight status in a longitudinal analysis in obese children before and after lifestyle intervention. BMC Pediatr 13:197.

Schepers R and Markus CR (2017) The interaction between 5-HTTLPR genotype and ruminative thinking on BMI. Br J Nutr 118:629–637.

Shinozaki G, Romanowicz M, Kung S, Rundell J and Mrazek D (2012) Investigation of serotonin transporter gene (SLC6A4) by child abuse history interaction with body mass index and diabetes mellitus of White female depressed psychiatric inpatients. Psychiatr Genet 22:109–114.

Southon A, Walder K, Sanigorski AM, Zimmet P, Nicholson GC, Kotowicz MA and Collier G (2003) The Taq IA and Ser311 Cys variants in the dopamine D2 receptor gene and obesity. Diabetes Nutr Metab 16:72–76.

Spitz MR, Detry MA, Pillow P, Hu YY, Amos CI, Hong WK and Wu X (2000) Variant alleles of the D2 dopamine receptor gene and obesity. Nutr Res 20:371–380.

Suriyaprom K, Phonrat B, Chuensumran U, Tungtrongchitr A and Tungtrongchitr R (2012) Association of HTTLPR and 5-HT_2A T102C variants with smoking characteristics and anthropometric profiles of Thai males. Genet Mol Res 11:4360–4369.

Thaler L, Groleau P, Badawi G, Sycz L, Zeramdini N, Too A, Israel M, Joober R, Bruce KR and Steiger H (2012) Epistatic interactions implicating dopaminergic genes in bulimia nervosa (BN): Relationships to eating-and personality-related psychopathology. Prog Neuropsychopharmacol Biol Psychiatry 39:120–128.

Thomas GN, Tomlinson B and Critchley JA (2000) Modulation of blood pressure and obesity with the dopamine D2 receptor gene TaqI variant. Hypertension 36:177–182.

Thompson PA, Shields PG, Freudenheim JL, Stone A, Vena JE, Marshall JR, Graham S, Laughlin R, Nemoto T, Kadlubar FF *et al.* (1998) Genetic variants in catechol-O-methyltransferase, menopausal status, and breast cancer risk. Cancer Res 58:2107–2110.

Tworoger SS, Chubak J, Aiello EJ, Yasui Y, Ulrich CM, Farin FM, Stapleton PL, Irwin ML, Potter JD, Schwartz RS *et al.* (2004) The effect of CYP19 and COMT variants on exercise-induced fat loss in postmenopausal women. Obes Res 12:972–981.

Uzun M, Saglar E, Kucukyildirim S, Erdem B, Unlu H and Mergen H (2015) Association of VNTR variants in DRD4, 5-HTT and DAT1 genes with obesity. Arch Physiol Biochem 121:75–79.

Valomon A, Holst SC, Bachmann V, Viola AU, Schmidt C, Zürcher J, Berger W, Cajochen C and Landolt HP (2014) Genetic variants of DAT1 and COMT differentially associate with actigraphy-derived sleep-wake cycles in young adults. Chronobiol Int 31:705–714.

Wallmeier D, Winkler JK, Fleming T, Woehning A, Huennemeyer K, Roeder E, Nawroth PP, Friederich HC, Wolfrum C, Schultz JH *et al.* (2013) Genetic modulation of the serotonergic pathway: Influence on weight reduction and weight maintenance. Genes Nutr 8:601–610.

Wang SS, Morton LM, Bergen AW, Lan EZ, Chatterjee N, Kvale P, Hayes RB, Chanock SJ and Caporaso NE (2007) Genetic variation in catechol-O-methyltransferase (COMT) and obesity in the prostate, lung, colorectal, and ovarian (PLCO) cancer screening trial. Hum Genet 122:41–49.

Wang SK, Lee YH, Kim JL and Chee IS (2014) No effect on body dissatisfaction of an interaction between 5-HTTLPR genotype and neuroticism in a young adult Korean population. Clin Psychopharmacol Neurosci 12:229–234.

Witte AV, Jansen S, Schirmacher A, Young P and Flöel A (2010) COMT Val158Met variant modulates cognitive effects of dietary intervention. Front Aging Neurosci 2:146.

Yeh J, Trang A, Henning SM, Wilhalme H, Carpenter C, Heber D and Li Z (2016) Food cravings, food addiction, and a dopamine-resistant (DRD2 A1) receptor variant in Asian American college students. Asia Pac J Clin Nutr 25:424–429.

Yim DS, Parkb SK, Yoo KY, Yoon KS, Chung HH, Kang HL, Ahn SH, Noh DY, Choe KJ, Jang IJ *et al.* (2001) Relationship between the Val158Met variant of catechol O-methyl transferase and breast cancer. Pharmacogenetics 11:279–286.

Yokum S, Marti CN, Smolen A and Stice E (2015) Relation of the multilocus genetic composite reflecting high dopamine signaling capacity to future increases in BMI. Appetite 87:38–45.

Zhang ZJ, Yao ZJ, Zhang XB, Chen JF, Sun J, Yao H, Hou G and Zhang XB (2003) No association of antipsychotic agent-induced weight gain with a DA receptor gene variant and therapeutic response. Acta Pharmacol Sin 24:235–240.
