# Supplementary material for: Influence of reward-related genetic variants on BMI and predisposition to obesity: Systematic review and meta-analysis
Source: Genet Mol Biol. 2026 May 22;49(Suppl 1):e20250216. doi: 10.1590/1678-4685-GMB-2025-0216 (PMC13196784; doi:10.1590/1678-4685-GMB-2025-0216)
Supplement: Table S4 - [file 1415-4757-GMB-49-s1-e20250216-s4.pdf]

Genetics and Molecular Biology
https://doi.org/10.1590/1678-4685-GMB-2025-0216

Supplementary Material to "Influence of reward-related genetic variants on BMI and predisposition to obesity: Systematic review and meta-analysis"

Table S4 – List of articles included in the systematic review.

#	Author, year	Reference	Country	Journal
1	Comings *et al.*, 1993	Comings DE, Flanagan SD, Dietz G, Muhleman D, Knell E and Gysin R (1993) The dopamine D2 receptor (DRD2) as a major gene in obesity and height. Biochem Med Metab Biol 50:176–185	USA	Biochemical Medicine and Metabolic Biology
2	Noble *et al.*, 1994	Noble EP, Noble RE, Ritchie T, Syndulko K, Bohlman MC, Noble LA, Zhang Y, Sparkes RS and Grandy DK (1994) D2 dopamine receptor gene and obesity. Int J Eat Disord 15:205–217.	USA	International Journal of Eating Disorders
3	Blum *et al.*, 1996	Blum K, Braverman ER, Wood RC, Gill J, Li C, Chen TJ, Taub M, Montgomery AR, Sheridan PJ and Cull JG (1996) Increased prevalence of the TaqI A1 allele of the dopamine receptor gene (DRD2) in obesity with comorbid substance use disorder: A preliminary report. Pharmacogenetics 6:297–305.	USA	Pharmacogenetics and Genomics
4	Lavigne *et al.*, 1997	Lavigne JA, Helzlsouer KJ, Huang HY, Strickland PT, Bell DA, Selmin O, Watson MA, Hoffman S, Comstock GW and Yager JD (1997) An association between the allele coding for a low activity variant of catechol-O-methyltransferase and the risk for breast cancer. Cancer Res 57:5493–5497	USA	Cancer Research
5	Poston 2nd *et al.*, 1998	Poston 2nd WS, Ericsson M, Linder J, Haddock CK, Hanis CL, Nilsson T, Aström M and Foreyt JP (1998) D4 dopamine receptor gene exon III variant and obesity risk. Eat Weight Disord 3:71–77	USA	Eating and Weight Disorders - Studies on Anorexia, Bulimia and Obesity
6	Thompson *et al.*, 1998	Thompson PA, Shields PG, Freudenheim JL, Stone A, Vena JE, Marshall JR,	USA	Cancer Research

#	Author, year	Reference	Country	Journal
		Graham S, Laughlin R, Nemoto T, Kadlubar FF *et al.* (1998) Genetic variants in catechol-O-methyltransferase, menopausal status, and breast cancer risk. Cancer Res 58:2107–2110.		
7	Millikan *et al.*, 1998	Millikan RC, Pittman GS, Tse CK, Duell E, Newman B, Savitz D, Moorman PG, Boissy RJ and Bell DA (1998) Catechol-O-methyltransferase and breast cancer risk. Carcinogenesis 19:1943–1947.	USA	Carcinogenesis
8	Spitz *et al.*, 2000	Spitz MR, Detry MA, Pillow P, Hu YY, Amos CI, Hong WK and Wu X (2000) Variant alleles of the D2 dopamine receptor gene and obesity. Nutr Res 20:371–380.	USA	Nutrition Research
9	Thomas *et al.*, 2000	Thomas GN, Tomlinson B and Critchley JA (2000) Modulation of blood pressure and obesity with the dopamine D2 receptor gene TaqI variant. Hypertension 36:177–182.	Hong Kong	Hypertension
10	Thomas *et al.*, 2001	Thomas GN, Critchley JAJH, Tomlinson B, Cockram CS and Chan JCN (2001) Relationships between the TaqI variant of the dopamine D2 receptor and blood pressure in hyperglycaemic and normoglycaemic Chinese subjects. Clin Endocrinol 55:605–611.	Hong Kong	Clinical Endocrinology
11	Mitrunen *et al.*, 2001	Mitrunen K, Jourenkova N, Kataja V, Eskelinen M, Kosma VM, Benhamou S, Kang D, Vainio H, Uusitupa M and Hirvonen A (2001) Polymorphic catechol-O-methyltransferase gene and breast cancer risk. Cancer Epidemiol Biomarkers Prev 10:635–640.	Finland	Cancer Epidemiology, Biomarkers & Prevention
12	Yim *et al.*, 2001	Yim DS, Parkb SK, Yoo KY, Yoon KS, Chung HH, Kang HL, Ahn SH, Noh DY, Choe KJ, Jang IJ *et al.* (2001) Relationship between the Val158Met variant of catechol O-methyl transferase and breast cancer. Pharmacogenetics 11:279–286.	South Korea	Pharmacogenetics and Genomics
13	Kocabas *et al.*, 2002	Kocabaş NA, Sardaş S, Cholerton S, Daly AK and Karakaya AE (2002) Cytochrome P450 CYP1B1 and catechol O-methyltransferase (COMT) genetic variants and breast cancer susceptibility in a Turkish population. Arch Toxicol 76:643–649.	Turkey	Archives of Toxicology

#	Author, year	Reference	Country	Journal
14	Epstein *et al.*, 2002	Epstein LH, Jaroni JL, Paluch RA, Leddy JJ, Vahue HE, Hawk L, Wileyto EP, Shields PG and Lerman C (2002) Dopamine transporter genotype as a risk factor for obesity in African-American smokers. Obes Res 10:1232–1240	USA	Obesity Research
15	Zhang *et al.*, 2003	Zhang ZJ, Yao ZJ, Zhang XB, Chen JF, Sun J, Yao H, Hou G and Zhang XB (2003) No association of antipsychotic agent-induced weight gain with a DA receptor gene variant and therapeutic response. Acta Pharmacol Sin 24:235–240.	China	Acta Pharmacologica Sinica
16	Hong *et al.*, 2003	Hong CC, Thompson HJ, Jiang C, Hammond GL, Tritchler D, Yaffe M and Boyd NF (2003) Val158Met variant in catechol-O-methyltransferase gene associated with risk factors for breast cancer. Cancer Epidemiol Biomarkers Prev 12:838–847	Canada	Cancer Epidemiology, Biomarkers & Prevention
17	Southon *et al.*, 2003	Southon A, Walder K, Sanigorski AM, Zimmet P, Nicholson GC, Kotowicz MA and Collier G (2003) The Taq IA and Ser311 Cys variants in the dopamine D2 receptor gene and obesity. Diabetes Nutr Metab 16:72–76.	Australia	Diabetes, Nutrition & Metabolism
18	Fang *et al.*, 2004	Fang YJ, Thomas GN, Xu ZL, Fang JQ, Critchley JA and Tomlinson B (2005) An affected pedigree member analysis of linkage between the dopamine D2 receptor gene TaqI variant and obesity and hypertension. Int J Cardiol 102:111–116.	Hong Kong	International Journal of Cardiology
19	Epstein *et al.*, 2004	Epstein LH, Wright SM, Paluch RA, Leddy JJ, Hawk LW Jr, Jaroni JL, Saad FG, Crystal-Mansour S, Shields PG and Lerman C (2004) Relation between food reinforcement and dopamine genotypes and its effect on food intake in smokers. Am J Clin Nutr 80:82–88.	USA	The American Journal of Clinical Nutrition
20	Camarena *et al.*, 2004	Camarena B, Santiago H, Aguilar A, Ruvinskis E, González-Barranco J and Nicolini H (2004) Family-based association study between the monoamine oxidase A gene and obesity: Implications for psychopharmacogenetic studies. Neuropsychobiology 49:126–129.	Mexico	Neuropsychobiology
21	Tworoger *et al.*, 2004	Tworoger SS, Chubak J, Aiello EJ, Yasui Y, Ulrich CM, Farin FM, Stapleton PL, Irwin ML, Potter JD, Schwartz RS *et al.* (2004) The effect of CYP19 and COMT variants on exercise-induced fat loss in postmenopausal women. Obes Res 12:972–981.	USA	Obesity Research

#	Author, year	Reference	Country	Journal
22	Kocabas *et al.*, 2005	Kocabaş NA, Sardaş S and Karakaya AE (2005) Variants related to estrogen and xenobiotic metabolism in healthy Turkish women. Arch Med Res 36:19–23.	Turkey	Archives of Medical Research
23	Munafò *et al.*, 2006	Munafò MR, Murphy MF and Johnstone EC (2006) Smoking cessation, weight gain, and DRD4−521 genotype. Am J Med Genet B Neuropsychiatr Genet 141:398–402	United Kindgom	American Journal of Medical Genetics
24	Need *et al.*, 2006	Need AC, Ahmadi KR, Spector TD and Goldstein DB (2006) Obesity is associated with genetic variants that alter dopamine availability. Ann Hum Genet 70:293–303	United Kindgom	Annals of Human Genetics
25	Morton *et al.*, 2006	Morton LM, Wang SS, Bergen AW, Chatterjee N, Kvale P, Welch R, Yeager M, Hayes RB, Chanock SJ and Caporaso NE (2006) DRD2 genetic variation in relation to smoking and obesity in the Prostate, Lung, Colorectal, and Ovarian Cancer Screening Trial. Pharmacogenet Genomics 16:901–910	USA	Pharmacogenetics and Genomics
26	Ducci *et al.*, 2006	Ducci F, Newman TK, Funt S, Brown GL, Virkkunen M and Goldman D (2006) A functional variant in the MAOA gene promoter (MAOA-LPR) predicts central dopamine function and body mass index. Mol Psychiatry 11:858–866	USA	Molecular Psychiatry
27	Epstein *et al.*, 2007	Epstein LH, Temple JL, Neaderhiser BJ, Salis RJ, Erbe RW and Leddy JJ (2007) Food reinforcement, the dopamine D2 receptor genotype, and energy intake in obese and nonobese humans. Behav Neurosci 121:877–886.	USA	Behavioral Neuroscience
28	Gorai *et al.*, 2007	Gorai I, Inada M, Morinaga H, Uchiyama Y, Yamauchi H, Hirahara F and Chaki O (2007) CYP17 and COMT gene variants can influence bone directly, or indirectly through their effects on endogenous sex steroids, in postmenopausal Japanese women. Bone 40:28–36.	Japan	Bone
29	Mergen *et al.*, 2007	Mergen H, Karaaslan C, Mergen M, Deniz Ozsoy E and Ozata M (2007) LEPR, ADBR3, IRS-1 and 5-HTT genes variants do not associate with obesity. Endocr J 54:89-94.	Turkey	Endocrine Journal
30	Chen *et al.*, 2007	Chen TJH, Blum K, Mathews D, Fisher L, Schnautz N and Braverman ER (2007) Reviewing the role of putative candidate genes in “Neurobesigenics”, a clinical	Taiwan	Gene Therapy and Molecular Biology

#	Author, year	Reference	Country	Journal
		subtype of Reward Deficiency Syndrome (RDS). Gene Ther Mol Biol 11:61–74.		
31	Wang *et al.*, 2007	Wang SS, Morton LM, Bergen AW, Lan EZ, Chatterjee N, Kvale P, Hayes RB, Chanock SJ and Caporaso NE (2007) Genetic variation in catechol-O-methyltransferase (COMT) and obesity in the prostate, lung, colorectal, and ovarian (PLCO) cancer screening trial. Hum Genet 122:41–49.	USA	Human Genetics
32	Nisoli *et al.*, 2007	Nisoli E, Brunani A, Borgomainerio E, Tonello C, Dioni L, Briscini L, Redaelli G, Molinari E, Cavagnini F and Carruba MO (2007) D2 dopamine receptor (DRD2) gene Taq1A variant and the eating-related psychological traits in eating disorders (anorexia nervosa and bulimia) and obesity. Eat Weight Disord 12:91–96.	Italy	Eating and Weight Disorders - Studies on Anorexia, Bulimia and Obesity
33	Annerbrink *et al.*, 2008	Annerbrink K, Westberg L, Nilsson S, Rosmond R, Holm G and Eriksson E (2008) Catechol O-methyltransferase val158-met variant is associated with abdominal obesity and blood pressure in men. Metabolism 57:708–711.	Sweden	Metabolism
34	Davis *et al.*, 2008	Davis C, Levitan RD, Kaplan AS, Carter J, Reid C, Curtis C, Patte K, Hwang R and Kennedy JL (2008) Reward sensitivity and the D2 dopamine receptor gene: A case-control study of binge eating disorder. Prog Neuropsychopharmacol Biol Psychiatry 32:620–628.	Canada	Progress in Neuro-Psychopharmacology and Biological Psychiatry
35	Sookoian *et al.*, 2008	Sookoian S, Gianotti TF, Gemma C, Burgueno A and Pirola CJ (2008) Contribution of the functional 5-HTTLPR variant of the SLC6A4 gene to obesity risk in male adults. Obesity 16:488–491	Argentina	Obesity
36	Brummet *et al.*, 2008	Brummett BH, Boyle SH, Siegler IC, Zuchner S, Ashley-Koch A and Williams RB (2008) Lipid levels are associated with a regulatory variant of the monoamine oxidase-A gene promoter (MAOA-uVNTR). Med Sci Monit 14:CR57–CR61.	USA	Medical Science Monitor
37	Eisenberg *et al.*, 2008	Eisenberg DT, Campbell B, Gray PB and Sorenson MD (2008) Dopamine receptor genetic variants and body composition in undernourished pastoralists: An exploration of nutrition indices among nomadic and recently settled Ariaal men of northern Kenya. BMC Evol Biol 8:173	USA	BMC Ecology and Evolution, formerly known as BMC Evolutionary Biology

#	Author, year	Reference	Country	Journal
38	Justenhoven *et al.*, 2008	Justenhoven C, Hamann U, Schubert F, Zapatka M, Pierl CB, Rabstein S, Selinski S, Mueller T, Ickstadt K, Gilbert M *et al.* (2008) Breast cancer: A candidate gene approach across the estrogen metabolic pathway. Breast Cancer Res Treat 137:49.	Germany	Breast Cancer Research and Treatment
39	Azzato *et al.*, 2009	Azzato EM, Morton LM, Bergen AW, Wang SS, Chatterjee N, Kvale P, Yeager M, Hayes RB, Chanock SJ and Caporaso NE (2009) SLC6A3 and body mass index in the Prostate, Lung, Colorectal and Ovarian Cancer Screening Trial. BMC Med Genet 10:9.	USA	BMC Medical Genetics
40	Lan *et al.*, 2009	Lan MY, Chang YY, Chen WH, Kao YF, Lin HS and Liu JS (2009) Serotonin transporter gene promoter variant is associated with body mass index and obesity in non-elderly stroke patients. J Endocrinol Invest 32:119–122.	Taiwan	Journal of Endocrinological Investigation
41	Lee *et al.*, 2009	Lee HY, Kim DJ, Lee HJ, Choi JE and Kim YK (2009) No association of serotonin transporter variant (5-HTTVNTR and 5-HTTLPR) with characteristics and treatment response to atypical antipsychotic agents in schizophrenic patients. Prog Neuropsychopharmacol Biol Psychiatry 33:276–280.	South Korea	Progress in Neuro-Psychopharmacology and Biological Psychiatry
42	Barnard *et al.*, 2009	Barnard ND, Noble EP, Ritchie T, Cohen J, Jenkins DJ, Turner-McGrievy G, Gloede L, Green AA and Ferdowsian H (2009) D2 dopamine receptor Taq1A variant, body weight, and dietary intake in type 2 diabetes. Nutrition 25:58–65.	USA	Nutrition
43	Kring *et al.*, 2009	Kring SI, Werge T, Holst C, Toubro S, Astrup A, Hansen T, Pedersen O and Sørensen TI (2009) Variants of serotonin receptor 2A and 2C genes and COMT in relation to obesity and type 2 diabetes. PLoS One 4:e6696.	Denmark	PLOS One
44	Davis *et al.*, 2009	Davis CA, Levitan RD, Reid C, Carter JC, Kaplan AS, Patte KA, King N, Curtis C and Kennedy JL (2009) Dopamine for "wanting" and opioids for "liking": A comparison of obese adults with and without binge eating. Obesity (Silver Spring) 17:1220–1225	Canada	Obesity
45	Gallicchio *et al.*, 2009	Gallicchio L, Chang HH, Christo DK, Thuita L, Huang HY, Strickland P, Ruczinski I, Clipp S and Helzlsouer KJ (2009) Single nucleotide variants in obesity-related	USA	BMC Medical Genetics

#	Author, year	Reference	Country	Journal
		genes and all-cause and cause-specific mortality: A prospective cohort study. BMC Med Genet 10:103.		
46	Linares *et al.*, 2010	Lloret Linares C, Hajj A, Poitou C, Simoneau G, Clement K, Laplanche JL, Lépine JP, Bergmann JF, Mouly S and Peoc'h K (2011) Pilot study examining the frequency of several gene variants involved in morphine pharmacodynamics and pharmacokinetics in a morbidly obese population. Obes Surg 21:1257–1264.	France	Obesity Surgery
47	Witte *et al.*, 2010	Witte AV, Jansen S, Schirmacher A, Young P and Flöel A (2010) COMT Val158Met variant modulates cognitive effects of dietary intervention. Front Aging Neurosci 2:146.	Germany	Frontiers in Aging Neuroscience
48	Iordanidou *et al.*, 2010	Iordanidou M, Tavridou A, Petridis I, Arvanitidis KI, Christakidis D, Vargemezis V and Manolopoulos VG (2010) The serotonin transporter promoter variant (5-HTTLPR) is associated with type 2 diabetes. Clin Chim Acta 411:167–171.	Greece	Clinica Chimica Acta
49	Bah *et al.*, 2010	Bah J, Westberg L, Baghaei F, Henningsson S, Rosmond R, Melke J, Holm G and Eriksson E (2010) Further exploration of the possible influence of variants in HTR2C and 5HTT on body weight. Metabolism 59:1156–1163.	Sweden	Metabolism
50	Levitan *et al.*, 2010	Levitan RD, Kaplan AS, Davis C, Lam RW and Kennedy JL (2010) A season-of-birth/DRD4 interaction predicts maximal body mass index in women with bulimia nervosa. Neuropsychopharmacology 35:1729–1733	Canada	Neuropsychopharmacology
51	Correia *et al.*, 2010	Correia CT, Almeida JP, Santos PE, Sequeira AF, Marques CE, Miguel TS, Abreu RL, Oliveira GG and Vicente AM (2010) Pharmacogenetics of risperidone therapy in autism: Association analysis of eight candidate genes with drug efficacy and adverse drug reactions. Pharmacogenomics J 10:418–430.	Turkey	Clinical and Experimental Hypertension
52	Epstein *et al.*, 2011	Epstein LH, Dearing KK and Erbe RW (2010) Parent-child concordance of Taq1 A1 allele predicts similarity of parent-child weight loss in behavioral family-based treatment programs. Appetite 55:363–366.	USA	Appetite
53	Tsuboi *et al.*, 2010	Tsuboi H, Sakakibara H, Yamakawa-Kobayashi K, Tatsumi A, Inamori T, Hamamoto R, Suzuki A and Shimoi K (2011) Val1483Ile variant in the fatty acid	Japan	Journal of Affective

#	Author, year	Reference	Country	Journal
		synthase gene was associated with depressive symptoms under the influence of psychological stress. J Affect Disord 134:448–452		Disorders
54	Cribb *et al.*, 2011	Cribb AE, Joy Knight M, Guernsey J, Dryer D, Hender K, Shawwa A, Tesch M and Saleh TM (2011) CYP17, catechol-O-methyltransferase, and glutathione transferase M1 genetic variants, lifestyle factors, and breast cancer risk in women on Prince Edward Island. Breast J 17:24–31.	Canada	The Breast Journal
55	Ariza *et al.*, 2012	Ariza M, Garolera M, Jurado MA, Garcia-Garcia I, Hernan I, Sánchez-Garre C, Vernet-Vernet M, Sender-Palacios MJ, Marques-Iturria I, Pueyo R *et al.* (2012) Dopamine genes (DRD2/ANKK1-TaqA1 and DRD4-7R) and executive function: Their interaction with obesity. PLoS One 7:e41482.	Spain	PLOS One
56	Markus *et al.*, 2012	Markus CR and Capello AE (2012) Contribution of the 5-HTTLPR gene by neuroticism on weight gain in male and female participants. Psychiatr Genet 22:279–285.	The Netherlands	Psychiatric Genetics
57	Chen *et al.*, 2012	Chen KC, Lin YC, Chao WC, Chung HK, Chi SS, Liu WS and Wu WT (2012) Association of genetic variants of glutamate decarboxylase 2 and the dopamine D2 receptor with obesity in Taiwanese subjects. Ann Saudi Med 32:121–126.	Taiwan	Annals of Saudi Medicine
58	Winkler *et al.*, 2012	Winkler JK, Woehning A, Schultz JH, Brune M, Beaton N, Challa TD, Minkova S, Roeder E, Nawroth PP, Friederich HC, Wolfrum C and Rudofsky G (2012) TaqIA variant in dopamine D2 receptor gene complicates weight maintenance in younger obese patients. Nutrition 28:996–1001.	Germany	Nutrition
59	Peralta-Leal *et al.*, 2012	Peralta-Leal V, Leal-Ugarte E, Meza-Espinoza JP, Dávalos-Rodríguez IP, Bocanegra-Alonso A, Acosta-González RI, Gonzales E, Nair S and Durán-González J (2012) Association of a serotonin transporter gene (SLC6A4) 5-HTTLPR variant with body mass index categories but not type 2 diabetes mellitus in Mexicans. Genet Mol Biol 35:589–593	Mexico	Human and Medical Genetics
60	Chen *et al.*, 2012	Chen AL, Blum K, Chen TJ, Giordano J, Downs BW, Han D, Barh D and Braverman ER (2012) Correlation of the Taq1 dopamine D2 receptor gene and percent body fat in obese and screened control subjects: A preliminary report. Food	Taiwan	Food & Function

#	Author, year	Reference	Country	Journal
		Funct 3:40–48		
61	Suriyaprom *et al.*, 2012	Suriyaprom K, Phonrat B, Chuensumran U, Tungtrongchitr A and Tungtrongchitr R (2012) Association of HTTLPR and 5-HT_2A T102C variants with smoking characteristics and anthropometric profiles of Thai males. Genet Mol Res 11:4360–4369.	Thailand	Genetics and Molecular Research
62	Markus *et al.*, 2012	Markus CR and Capello AEM (2012) Contribution of the 5-HTTLPR gene by neuroticism on weight gain in male and female participants. Psychiatr Genet 22:279–285	The Netherlands	Psychiatric Genetics
63	Hill *et al.*, 2012	Hill LD, Ewens KG, Maher BS, York TP, Legro RS, Dunaif A and Strauss JF 3rd (2012) Catechol-O-methyltransferase (COMT) single nucleotide variants and haplotypes are not major risk factors for polycystic ovary syndrome. Mol Cell Endocrinol 350:72–77.	USA	Molecular and Cellular Endocrinology
64	Shinozaki *et al.*, 2012	Shinozaki G, Romanowicz M, Kung S, Rundell J and Mrazek D (2012) Investigation of serotonin transporter gene (SLC6A4) by child abuse history interaction with body mass index and diabetes mellitus of white female depressed psychiatric inpatients. Psychiatr Genet 22:109–114.	USA	Psychiatric Genetics
65	Thaler *et al.*, 2012	Thaler L, Groleau P, Badawi G, Sycz L, Zeramdini N, Too A, Israel M, Joober R, Bruce KR and Steiger H (2012) Epistatic interactions implicating dopaminergic genes in bulimia nervosa (BN): Relationships to eating-and personality-related psychopathology. Prog Neuropsychopharmacol Biol Psychiatry 39:120–128.	Canada	Progress in Neuro-Psychopharmacology and Biological Psychiatry
66	Cameron *et al.*, 2013	Cameron JD, Riou ME, Tesson F, Goldfield GS, Rabasa-Lhoret R, Brochu M *et al.* (2013) The TaqIA RFLP is associated with attenuated intervention-induced body weight loss and increased carbohydrate intake in post-menopausal obese women. Appetite 60:111–116	Canada	Appetite
67	Wallmeier *et al.*, 2013	Wallmeier D, Winkler JK, Fleming T, Woehning A, Huennemeyer K, Roeder E, Nawroth PP, Friederich HC, Wolfrum C, Schultz JH *et al.* (2013) Genetic modulation of the serotonergic pathway: Influence on weight reduction and weight	Germany	Genes & Nutrition

#	Author, year	Reference	Country	Journal
		maintenance. Genes Nutr 8:601–610.		
68	Shinozaki *et al.*, 2013	Shinozaki G, Romanowicz M, Kung S, Rundell J and Mrazek D (2012) Investigation of serotonin transporter gene (SLC6A4) by child abuse history interaction with body mass index and diabetes mellitus of White female depressed psychiatric inpatients. Psychiatr Genet 22:109–114.	USA	Journal of Affective Disorders
69	Carpenter *et al.*, 2013	Carpenter CL, Wong AM, Li Z, Noble EP and Heber D (2013) Association of dopamine D2 receptor and leptin receptor genes with clinically severe obesity. Obesity (Silver Spring) 21:E467–E473	USA	Obesity
70	Roth *et al.*, 2013	Roth CL, Hinney A, Schur EA, Elfers CT and Reinehr T (2013) Association analyses for dopamine receptor gene variants and weight status in a longitudinal analysis in obese children before and after lifestyle intervention. BMC Pediatr 13:197.	USA	BMC Pediatrics
71	Sikora *et al.*, 2013	Sikora M, Gese A, Czypicki R, Gąsior M, Tretyn A, Chojnowski J, Bieliński M, Jaracz M, Kamińska A, Junik R, Borkowska A (2013) Correlations between variants in genes coding elements of dopaminergic pathways and body mass index in overweight and obese women. Endokrynol Pol 64:101–107.	Poland	Endokrynologia Polska
72	Capello *et al.*, 2014	Capello AE and Markus CR (2014) Differential influence of the 5-HTTLPR genotype, neuroticism and real-life acute stress exposure on appetite and energy intake. Appetite 77:83–93.	The Netherlands	Appetite
73	Wang *et al.*, 2014	Wang SK, Lee YH, Kim JL and Chee IS (2014) No effect on body dissatisfaction of an interaction between 5-HTTLPR genotype and neuroticism in a young adult Korean population. Clin Psychopharmacol Neurosci 12:229–234.	South Korea	Clinical Psychopharmacology and Neuroscience
74	Hursel *et al.*, 2014	Hursel R, Janssens PL, Bouwman FG, Mariman EC and Westerterp-Plantenga MS (2014) The role of catechol-O-methyl transferase Val(108/158)Met variant (rs4680) in the effect of green tea on resting energy expenditure and fat oxidation: A pilot study. PLoS One 9:e106220.	The Netherlands	PLoS One

#	Author, year	Reference	Country	Journal
75	Valomon *et al.*, 2014	Valomon A, Holst SC, Bachmann V, Viola AU, Schmidt C, Zürcher J, Berger W, Cajochen C and Landolt HP (2014) Genetic variants of DAT1 and COMT differentially associate with actigraphy-derived sleep-wake cycles in young adults. Chronobiol Int 31:705–714.	Switzerland	Chronobiology International
76	Capello *et al.*, 2014	Capello AE and Markus CR (2014) Effect of sub chronic tryptophan supplementation on stress-induced cortisol and appetite in subjects differing in 5-HTTLPR genotype and trait neuroticism. Psychoneuroendocrinology 45:96–107.	The Netherlands	Psychoneuroendocrinology
77	Athanasoulia *et al.*, 2014	Athanasoulia AP, Sievers C, Uhr M, Ising M, Stalla GK and Schneider HJ (2014) The effect of the ANKK1/DRD2 Taq1A variant on weight changes of dopaminergic treatment in prolactinomas. Pituitary 17:240–245.	Germany	Pituitary
78	Rob Markus *et al.*, 2014	Markus CR, Jonkman LM, Capello A, Leinders S and Hüsch F (2015) Sucrose preload reduces snacking after mild mental stress in healthy participants as a function of 5-hydroxytryptamine transporter gene promoter variant. Stress 18:149–159.	The Netherlands	The International Journal on the Biology of Stress
79	Fang *et al.*, 2014	Yang F, Chen XD, Tan LJ, Shen J, Li DY, Zhang F, Sha BY and Deng HW (2014) Genome wide association study: Searching for genes underlying body mass index in the Chinese. Biomed Environ Sci 27:360–370. doi:10.3967/bes2014.061	China	Biomedical and Environmental Sciences
80	Hameed *et al.*, 2015	Hameed A, Ajmal M, Nasir M and Ismail M (2015) Genetic association analysis of serotonin transporter variant (5-HTTLPR) with type 2 diabetes patients of Pakistani population. Diabetes Res Clin Pract 108:67–71.	Pakistan	Diabetes Research and Clinical Practice
81	Uzun *et al.*, 2015	Uzun M, Saglar E, Kucukyildirim S, Erdem B, Unlu H and Mergen H (2015) Association of VNTR variants in DRD4, 5-HTT and DAT1 genes with obesity. Arch Physiol Biochem 121:75–79.	Turkey	Archives of Physiology and Biochemistry
82	Dias *et al.*, 2015	Dias H, Muc M, Padez C and Manco L (2016) Association of variants in 5-HTT (SLC6A4) and MAOA genes with measures of obesity in young adults of Portuguese origin. Arch Physiol Biochem 122:8–13.	Portugal	Archives of Physiology and Biochemistry

#	Author, year	Reference	Country	Journal
83	Kvaløy *et al.*, 2015	Kvaløy K, Holmen J, Hveem K and Holmen TL (2015) Genetic effects on longitudinal changes from healthy to adverse weight and metabolic status—the HUNT study. PLoS One 10:e0139632	Norway	PLoS One
84	Borkowska *et al.*, 2015	Borkowska A, Bieliński M, Szczęsny W, Szwed K, Tomaszewska M, Kałwa A, Lesiewska N, Junik R, Gołębiewski M, Sikora M *et al.* (2015) Effect of the 5-HTTLPR variant on affective temperament, depression and body mass index in obesity. J Affect Disord 184:193–197.	Poland	Journal of Affective Disorders
85	Yokum *et al.*, 2015	Yokum S, Marti CN, Smolen A and Stice E (2015) Relation of the multilocus genetic composite reflecting high dopamine signaling capacity to future increases in BMI. Appetite 87:38–45.	USA	Appetite
86	Yeh *et al.*, 2016	Yeh J, Trang A, Henning SM, Wilhalme H, Carpenter C, Heber D and Li Z (2016) Food cravings, food addiction, and a dopamine-resistant (DRD2 A1) receptor variant in Asian American college students. Asia Pac J Clin Nutr 25:424–429.	USA	Asia Pacific Journal of Clinical Nutrition
87	Jawinski *et al.*, 2016	Jawinski P, Tegelkamp S, Sander C, Häntzsch M, Huang J, Mauche N, Scholz M, Spada J, Ulke C, Burkhardt R *et al.* (2016) Time to wake up: No impact of COMT Val158Met gene variation on circadian preferences, arousal regulation and sleep. Chronobiol Int 33:893–905.	Germany	Chronobiology International
88	Hinderberger *et al.*, 2016	Hinderberger P, Rullmann M, Drabe M, Luthardt J, Becker GA, Blüher M, Regenthal R, Sabri O and Hesse S (2016) The effect of serum BDNF levels on central serotonin transporter availability in obese versus non-obese adults: A [(11)C]DASB positron emission tomography study. Neuropharmacology 110:530–536.	Germany	Neuropharmacology
89	Yadav *et al.*, 2016	Yadav S, Devi NM, Singh HJ and Saraswathy KN (2016) Dopamine receptor D2 gene variant and interaction with the body mass index: A study among two tribal populations of Central India. Gene Rep 4:269–271.	India	Gene Reports
90	Bieliński *et al.*, 2017	Bieliński M, Jaracz M, Lesiewska N, Tomaszewska M, Sikora M, Junik R, Kamińska A, Tretyn A and Borkowska A (2017) Association between COMT Val158Met and DAT1 variants and depressive symptoms in the obese population.	Poland	Neuropsychiatric Disease and Treatment

#	Author, year	Reference	Country	Journal
		Neuropsychiatr Dis Treat 13:2221–2229.		
91	González-Giraldo *et al.*, 2017	González-Giraldo Y, Trujillo ML and Forero DA (2018) Two dopaminergic genes, DRD4 and SLC6A3, are associated with body mass index in a Colombian sample of young adults. Arch Physiol Biochem 124:330–334.	Colombia	Archives of Physiology and Biochemistry
92	Pedram *et al.*, 2017	Pedram P, Zhai G, Gulliver W, Zhang H and Sun G (2017) Two novel candidate genes identified in adults from the Newfoundland population with addictive tendencies towards food. Appetite 115:71–79	Canada	Appetite
93	Schepers *et al.*, 2017	Schepers R and Markus CR (2017) The interaction between 5-HTTLPR genotype and ruminative thinking on BMI. Br J Nutr 118:629–637.	The Netherlands	British Journal of Nutrition
94	Lek *et al.*, 2018	Lek FY, Ong HH and Say YH (2018) Association of dopamine receptor D2 gene (DRD2) Taq1 variants with eating behaviors and obesity among Chinese and Indian Malaysian university students. Asia Pac J Clin Nutr 27:707–717	Malaysia	Asia Pacific Journal of Clinical Nutrition
95	Rivera-Iñiguez *et al.*, 2018	Rivera-Iñiguez I, Panduro A, Ramos-Lopez O, Villaseñor-Bayardo SJ and Roman S (2019) DRD2/ANKK1 TaqI A1 variant associates with overconsumption of unhealthy foods and biochemical abnormalities in a Mexican population. Eat Weight Disord 24:835–844.	Mexico	Eating and Weight Disorders - Studies on Anorexia, Bulimia and Obesity
96	Frank *et al.*, 2018	Frank GKW, Shott ME, DeGuzman MC and Smolen A (2018) Dopamine D2 -141C Ins/Del and Taq1A variants, body mass index, and prediction error brain response. Transl Psychiatry 8:102.	USA	Translational Psychiatry
97	Palacios *et al.*, 2018	Palacios A, Canto P, Tejeda ME, Stephano S, Luján H, García-García E, Rojano-Mejía D and Méndez JP (2018) Complete sequence of the ANKK1 gene in Mexican-Mestizo individuals with obesity, with or without binge eating disorder. Eur Psychiatry 54:59–64.	Mexico	European Psychiatry
98	Pavlova *et al.*, 2019	Pavlova NI, Kurtanov KA, Diakonova AT, Solovyeva NA, Sydykova LA, Aleksandrova TN and Solovyeva YA (2019) Interrelation of the FTO rs9939609 SNP and the DAT1 rs27072 SNP with body mass index and degree of obesity in the	Russia	International Journal of Biomedicine

#	Author, year	Reference	Country	Journal
		population of Yakuts. Int J Biomed 9:210–215		
99	Ramos-Lopez *et al.*, 2019	Ramos-Lopez O, Mejia-Godoy R, Frías-Delgadillo KJ, Torres-Valadez R, Flores-García A, Sánchez-Enríquez S, Aguiar-García P, Martínez-López E and Zepeda-Carrillo EA (2019) Interactions between DRD2/ANKK1 TaqIA variant and dietary factors influence plasma triglyceride concentrations in diabetic patients from Western Mexico: A cross-sectional study. Nutrients 11:2863.	Mexico	Nutrients
100	Mehri *et al.*, 2019	Mehri F, Tahmasebi Fard Z and Ghoraeian P (2019) The investigation of functional genetic variation in COMT gene promoter (rs2020917 & rs2075507) in Iranian patients with breast cancer. Int J Cancer Manag 12:e92008	Iran	International Journal of Cancer Management
101	Asadzadeh *et al.*, 2019	Asadzadeh A, Ghaheh HSS, Sholehvar F, Takhshid M and Naghizadeh MM (2019) Investigation of the association between 5-hydroxytryptamine transporter gene-linked polymorphic region with type 2 diabetes mellitus, obesity and biochemical profiles of serum in Iranian population. Avicenna J Med Biotechnol 11:239	Iran	Avicenna Journal of Medical Biotechnology
102	Lim *et al.*, 2020	Lim ZM, Chie QT and Teh LK (2020) Influence of dopamine receptor gene on eating behaviour and obesity in Malaysia. Meta Gene 25:100736.	Malaysia	Meta Gene
103	Galaviz-Hernández *et al.*, 2020	Galaviz-Hernández C, Lazalde-Ramos BP, Martínez-Cortés G, Rangel-Villalobos H, Martínez-Aguilar G, Leal-Ugarte E, Peralta-Leal V *et al.* (2020) Association of the 5HTTLPR variant with obesity in Mexican women with high Native American ancestry. Genet Test Mol Biomarkers 24:754–758	Mexico	Genetic Testing and Molecular Biomarkers
104	Gassó *et al.*, 2020	Gassó P, Arnaiz JA, Mas S, Lafuente A, Bioque M, Cuesta MJ, Díaz-Caneja CM, García C, Lobo A, González-Pinto A *et al.* (2020) Association study of candidate genes with obesity and metabolic traits in antipsychotic-treated patients with first-episode psychosis over a 2-year period. J Psychopharmacol 34:514–523.	Spain	Journal of Psychopharmacology
105	Aliasghari *et al.*, 2021	Aliasghari F, Mahdavi R, Barati M, Nazm SA, Yasari S, Bonyadi M and Jabbari M (2021) Genotypes of ANKK1 and DRD2 genes and risk of metabolic syndrome and its components: A cross-sectional study on Iranian women. Obes Res Clin Pract 15:449–454.	Iran	Obesity Research & Clinical Practice

#	Author, year	Reference	Country	Journal
106	Aliasghari *et al.*, 2021	Aliasghari F, Pirdehghan A, Aghamohammadzadeh N, Rashtchizadeh N, Azarfam P and Yaghmaei P (2021) Associations of the ANKK1 and DRD2 gene variants with overweight, obesity and hedonic hunger among women from the Northwest of Iran. Eat Weight Disord 26:305–312	Iran	Eating and Weight Disorders - Studies on Anorexia, Bulimia and Obesity
107	Paderina *et al.*, 2021	Paderina DZ, Boiko AS, Pozhidaev IV, Bocharova AV, Mednova IA, Fedorenko OY, Kornetova EG, Loonen AJM, Semke AV, Bokhan NA *et al.* (2021) Genetic variants of 5-HT receptors and antipsychotic-induced metabolic dysfunction in patients with schizophrenia. J Pers Med 11:181.	Russia	Journal of Personalized Medicine
108	Matsunaga *et al.*, 2021	Matsunaga M, Ohtsubo Y, Masuda T, Noguchi Y, Yamasue H and Ishii K (2021) A genetic variation in the Y chromosome among modern Japanese males related to several physiological and psychological characteristics. Front Behav Neurosci 15:774879.	Japan	Frontiers in Behavioral Neuroscience
109	Beyer *et al.*, 2021	Beyer F, Zhang R, Scholz M, Wirkner K, Loeffler M, Stumvoll M, Villringer A and Witte AV (2021) Higher BMI, but not obesity-related genetic variants, correlates with lower structural connectivity of the reward network in a population-based study. Int J Obes (Lond) 45:491–501.	Germany	International Journal of Obesity
110	Obregón *et al.*, 2022	Obregón AM, Oyarce K, García-Robles MA, Valladares M, Pettinelli P and Goldfield GS (2022) Association of the dopamine D2 receptor rs1800497 variant with food addiction, food reinforcement, and eating behavior in Chilean adults. Eat Weight Disord 27:215–224.	Chile	Eating and Weight Disorders - Studies on Anorexia, Bulimia and Obesity
111	Bednarova *et al.*, 2023	Bednarova A, Habalova V, Iannaccone SF, Tkac I, Jarcuskova D, Krivosova M, Marcatili M and Hlavacova N (2023) Association of HTTLPR, BDNF, and FTO genetic variants with completed suicide in Slovakia. J Pers Med 13:501.	Slovakia	Journal of Personalized Medicine
112	Arrue *et al.*, 2023	Arrue A, Olivas O, Erkoreka L, Alvarez FJ, Arnaiz A, Varela N, Bilbao A, Rodríguez JJ, Moreno-Calle MT and Gordo E (2023) Multilocus genetic profile reflecting low dopaminergic signaling is directly associated with obesity and cardiometabolic disorders due to antipsychotic treatment. Pharmaceutics 15:2134.	Spain	Pharmaceutics

#	Author, year	Reference	Country	Journal
113	Daza-Hernández *et al.*, 2023	Daza-Hernández S, Pérez-Luque E, Martínez-Cordero C, Figueroa-Vega N, Cardona-Alvarado MI and Muñoz-Montes N (2023) Analysis of factors associated with outcomes of bariatric surgery: rs1800497 ANKK1, rs1799732 DRD2 genetic variants, eating behavior, hedonic hunger, and depressive symptoms. J Gastrointest Surg 27:1778-1784.	Mexico	Journal of Gastrointestinal Surgery
114	Hidalgo Vira *et al.*, 2023	Hidalgo Vira N, Oyarce K, Valladares Vega M, Goldfield GS, Guzmán-Gutiérrez E and Obregón AM (2023) No association of the dopamine D2 receptor genetic bilocus score (rs1800497/rs1799732) on food addiction and food reinforcement in Chilean adults. Front Behav Neurosci 17:1067384.	Chile	Frontiers in Behavioral Neuroscience
115	Losada-Casallas *et al.*, 2024	Losada-Casallas K, Cepeda-Leal I, Ruiz N, Muñoz-Ospina B and Ortega-Avila G (2024) Body index mass not associated with DRD4, DAT1, BDNF, and COMT gene variants in young adults without depression or anxiety disorders. Genet Mol Res 23:1–10.	Colombia	Genetics and Molecular Research
116	Markus; Keulers, 2025	Markus CR and Keulers EHH (2025) The serotonin gene 5-HTTLPR and brain food-reward responses during sadness: A mood-induction neuroimaging study. J Affect Disord 384:1–11.	The Netherlands	Journal of Affective Disorders
117	Yatsuda *et al.*, 2025	Yatsuda M, Furou M, Kamachi K, Sakamoto K, Shoji K, Ishihara O and Kagawa Y (2025) Serotonin transporter gene variants predict adherence to weight loss programs independently of obesity-related genes. Nutrients 17:1094.	Japan	Nutrients
