# Supplementary material for: Influence of reward-related genetic variants on BMI and predisposition to obesity: Systematic review and meta-analysis
Source: Genet Mol Biol. 2026 May 22;49(Suppl 1):e20250216. doi: 10.1590/1678-4685-GMB-2025-0216 (PMC13196784; doi:10.1590/1678-4685-GMB-2025-0216)
Supplement: Table S5 - [file 1415-4757-GMB-49-s1-e20250216-s5.pdf]

Genetics and Molecular Biology
https://doi.org/10.1590/1678-4685-GMB-2025-0216

Supplementary Material to "Influence of reward-related genetic variants on BMI and predisposition to obesity: Systematic review and meta-analysis"

Table S5 – Risk of bias for each included study.

#	Study	Title	1	2	3	4	5	6	7	8	9	10	11	Total	Q-Genie classification
1	Comings *et al.*, 1993	The Dopamine D2 Receptor (DRD2) as a Major Gene in Obesity and Height	6	6	5	4	3	6	6	4	4	5	5	54	Good quality
2	Noble *et al.*, 1994	Dopamine Receptor Gene and Obesity	6	6	5	4	3	6	5	4	4	5	5	53	Good quality
3	Blum *et al.*, 1996	Increased prevalence of the Taq I A1allele of the dopamine receptor gene (DRD2) in obesity with comorbid substance use disorder: A preliminary report	6	6	5	4	3	6	6	4	4	5	5	54	Good quality
4	Lavigne *et al.*, 1997	An association between the allele coding for a low activity variant of catechol-O-methyltransferase and the risk for breast cancer	6	6	4	6	5	5	4	3	4	4	5	52	Good quality
5	Poston 2nd *et al.*, 1998	D4 dopamine receptor gene exon III variant and obesity risk	6	6	5	6	6	5	6	5	4	6	6	61	Good quality
6	Thompson *et al.*, 1998	Genetic variants in catechol-O-methyltransferase, menopausal status, and breast cancer risk	6	6	6	6	6	5	5	4	5	4	6	59	Good quality
7	Millikan *et al.*, 1998	Catechol-O-methyltransferase and breast cancer risk	6	6	6	6	6	6	6	5	6	5	6	64	Good quality
8	Spitz *et al.*, 2000	Variant alleles of the D2 dopamine receptor gene and obesity	6	6	6	4	4	5	6	6	6	6	5	60	Good quality
9	Thomas *et al.*, 2000	Modulation of Blood Pressure and Obesity With the Dopamine D2 Receptor Gene TaqI Variant	6	6	6	5	5	6	6	6	6	6	6	64	Good quality
10	Thomas *et al.*, 2001	Relationships between the TaqI variant of the dopamine D2 receptor and blood pressure in hyperglycaemic and normoglycaemic Chinese subjects	6	6	6	7	5	6	6	6	6	6	6	66	Good quality
11	Mitrunen *et al.*, 2001	Polymorphic Catechol-O-methyltransferase Gene and Breast Cancer Risk	6	5	6	6	5	5	6	6	6	6	6	63	Good quality

#	Study	Title	1	2	3	4	5	6	7	8	9	10	11	Total	Q-Genie classification
12	Yim *et al.*, 2001	Relationship between the Val158Met variant of catechol O-methyl transferase and breast cancer	6	6	6	4	5	6	4	5	5	5	6	58	Good quality
13	Kocabaş *et al.*, 2002	Cytochrome P450 CYP1B1 and catechol O-methyltransferase (COMT) genetic variants and breast cancer susceptibility in a Turkish population	6	6	6	4	5	6	4	5	5	5	6	58	Good quality
14	Epstein *et al.*, 2002	Dopamine transporter genotype as a risk factor for obesity in African-American smokers	6	6	6	5	5	6	5	6	6	6	6	63	Good quality
15	Zhang *et al.*, 2003	No association of antipsychotic agent-induced weight gain with a DA receptor gene variant and therapeutic response	6	5	5	4	5	6	4	5	5	5	6	56	Good quality
16	Hong *et al.*, 2003	Val158Met Variant in Catechol-O-methyltransferase Gene Associated with Risk Factors for Breast Cancer	6	5	6	6	5	5	6	6	6	6	6	63	Good quality
17	Southon *et al.*, 2003	The Taq IA and Ser311 Cys variants in the dopamine D2 receptor gene and obesity	6	6	6	4	5	6	4	5	5	6	6	59	Good quality
18	Fang *et al.*, 2005	An affected pedigree member analysis of linkage between the dopamine D2 receptor gene TaqI variant and obesity and hypertension	6	6	6	5	5	6	5	6	6	6	6	63	Good quality
19	Epstein *et al.*, 2004	Relation between food reinforcement and dopamine genotypes and its effect on food intake in smokers	6	6	6	5	5	6	5	6	6	6	6	63	Good quality
20	Camarena *et al.*, 2004	Family-based association study between the monoamine oxidase A gene and obesity: Implications for psychopharmacogenetic studies	6	5	5	4	5	5	4	5	5	6	5	55	Good quality
21	Tworoger *et al.*, 2004	The effect of CYP19 and COMT variants on exercise-induced fat loss in postmenopausal women	6	6	6	5	6	6	6	6	6	6	6	65	Good quality
22	Kocabaş *et al.*, 2005	Variants related to estrogen and xenobiotic metabolism in healthy Turkish women	6	5	5	5	5	5	4	5	5	5	5	55	Good quality
23	Munafò *et al.*, 2006	Smoking cessation, weight gain, and DRD4 −521 genotype	6	5	6	6	6	6	5	6	6	6	6	64	Good quality
24	Need *et al.*, 2006	Obesity is associated with genetic variants that alter dopamine availability	6	6	6	7	6	6	6	6	6	6	6	67	Good quality
25	Morton *et al.*, 2006	DRD2 genetic variation in relation to smoking and obesity in the Prostate, Lung, Colorectal, and Ovarian Cancer Screening Trial	6	6	6	7	6	6	6	6	6	6	6	67	Good quality
26	Ducci *et al.*, 2006	A functional variant in the MAOA gene promoter (MAOA-LPR) predicts central dopamine function and body mass index	6	5	6	6	5	6	5	6	6	6	6	63	Good quality
27	Epstein *et al.*,	Food Reinforcement, the Dopamine D2 Receptor Genotype,	6	6	6	5	5	6	5	6	6	6	6	63	Good quality

#	Study	Title	1	2	3	4	5	6	7	8	9	10	11	Total	Q-Genie classification
	2007	and Energy Intake in Obese and Nonobese Humans													
28	Gorai *et al.*, 2007	CYP17 and COMT gene variants can influence bone directly, or indirectly through their effects on endogenous sex steroids, in postmenopausal Japanese women	6	6	6	6	6	6	5	6	6	6	6	65	Good quality
29	Mergen *et al.*, 2007	LEPR, ADBR3, IRS-1 and 5-HTT genes variants do not associate with obesity	6	6	6	5	5	6	5	6	6	6	6	63	Good quality
30	Chen *et al.*, 2007	Reviewing the role of putative candidate genes in "Neurobesigenics," a clinical subtype of Reward Deficiency Syndrome (RDS)	6	6	6	5	5	6	5	6	6	6	6	63	Good quality
31	Nisoli *et al.*, 2007	D2 dopamine receptor (DRD2) gene Taq1A variant and the eatingrelated psychological traits in eating disorders (anorexia nervosa and bulimia) and obesity	7	6	6	7	7	5	4	6	6	6	6	66	Good quality
32	Wang *et al.*, 2007	Genetic variation in catechol-O-methyltransferase (COMT) and obesity in the prostate, lung, colorectal, and ovarian (PLCO) cancer screening trial	6	6	6	4	5	6	5	6	6	6	6	62	Good quality
33	Annerbrink *et al.*, 2008	Catechol O-methyltransferase val158-met variant is associated with abdominal obesity and blood pressure in men	6	5	7	5	7	7	4	5	5	6	6	63	Good quality
34	Davis *et al.*, 2008	Reward sensitivity and the D2 dopamine receptor gene: A case-control study of binge eating disorder	6	6	6	4	5	6	5	6	6	6	6	62	Good quality
35	Sookoian *et al.*, 2008	Contribution of the Functional 5-HTTLPR Variant of the SLC6A4 Gene to Obesity Risk in Male Adults	6	6	6	7	7	6	7	7	6	7	7	72	Good quality
36	Brummet *et al.*, 2008	Lipid levels are associated with a regulatory variant of the monoamine oxidase-A gene promoter (MAOA-uVNTR)	6	6	5	6	6	7	6	6	4	6	6	64	Good quality
37	Eisenberg *et al.*, 2008	Dopamine receptor genetic variants and body composition in undernourished pastoralists: An exploration of nutrition indices among nomadic and recently settled Ariaal men of northern Kenya	6	6	5	5	6	7	5	6	6	6	6	64	Good quality
38	Justenhoven *et al.*, 2008	Breast cancer: A candidate gene approach across the estrogen metabolic pathway	6	7	7	6	7	6	5	6	6	6	6	68	Good quality
39	Azzato *et al.*, 2009	SLC6A3 and body mass index in the Prostate, Lung, Colorectal and Ovarian Cancer Screening Trial	7	7	7	7	7	7	6	6	6	7	7	74	Good quality
40	Lan *et al.*, 2009	Serotonin transporter gene promoter variant is associated with body mass index and obesity in non-elderly stroke patients	6	6	6	6	6	6	5	6	6	6	6	65	Good quality
41	Lee *et al.*, 2009	No association of serotonin transporter variant (5-	6	6	6	6	6	6	5	6	6	6	6	65	Good quality

#	Study	Title	1	2	3	4	5	6	7	8	9	10	11	Total	Q-Genie classification
		HTTVNTR and 5-HTTLPR) with characteristics and treatment response to atypical antipsychotic agents in schizophrenic patients													
42	Barnard *et al.*, 2009	D2 dopamine receptor Taq1A variant, body weight, and dietary intake in type 2 diabetes	6	6	6	6	6	6	5	6	6	6	6	65	Good quality
43	Kring *et al.*, 2009	Variants of Serotonin Receptor 2A and 2C Genes and COMT in Relation to Obesity and Type 2 Diabetes	6	6	6	6	6	6	5	6	6	6	6	65	Good quality
44	Davis *et al.*, 2009	Dopamine for "Wanting" and Opioids for "Liking": A Comparison of Obese Adults With and Without Binge Eating	6	6	6	6	6	6	5	6	6	6	6	65	Good quality
45	Gallicchio *et al.*, 2009	Single nucleotide variants in obesity-related genes and all-cause and cause-specific mortality: A prospective cohort study	6	6	6	6	6	6	5	6	6	6	6	65	Good quality
46	Lloret Linares *et al.*, 2011	Pilot Study Examining the Frequency of Several Gene Variants Involved in Morphine Pharmacodynamics and Pharmacokinetics in a Morbidly Obese Population	6	6	6	6	6	6	5	6	6	6	6	65	Good quality
47	Witte *et al.*, 2010	COMT Val158Met Variant Modulates Cognitive Effects of Dietary Intervention	6	6	6	6	6	6	5	6	6	6	6	65	Good quality
48	Iordanidou *et al.*, 2010	The serotonin transporter promoter variant (5-HTTLPR) is associated with type 2 diabetes	6	6	6	6	6	6	5	6	6	6	6	65	Good quality
49	Bah *et al.*, 2010	Further exploration of the possible influence of variants in HTR2C and 5HTT on body weight	6	6	6	6	6	6	5	6	6	6	6	65	Good quality
50	Levitan *et al.*, 2010	A Season-of-Birth/DRD4 Interaction Predicts Maximal Body Mass Index in Women with Bulimia Nervosa	6	6	6	6	6	6	5	6	6	6	6	65	Good quality
51	Tsuboi *et al.*, 2011	Val1483Ile variant in the fatty acid synthase gene was associated with depressive symptoms under the influence of psychological stress	6	6	6	6	6	6	5	6	6	6	6	65	Good quality
52	Epstein *et al.*, 2011	Parent–child concordance of Taq1 A1 allele predicts similarity of parent–child weight loss in behavioral family-based treatment programs	6	6	6	6	6	6	5	6	6	6	6	65	Good quality
53	Correia *et al.*, 2010	Pharmacogenetics of risperidone therapy in autism: Association analysis of eight candidate genes with drug efficacy and adverse drug reactions	5	5	5	4	5	5	4	5	5	4	5	52	Good quality
54	Cribb *et al.*, 2011	CYP17, catechol-o-methyltransferase, and glutathione transferase M1 genetic variants, lifestyle factors, and breast cancer risk in women on Prince Edward Island	6	6	6	5	6	6	5	6	6	6	6	64	Good quality

#	Study	Title	1	2	3	4	5	6	7	8	9	10	11	Total	Q-Genie classification
55	Ariza *et al.*, 2012	Dopamine Genes (DRD2/ANKK1-TaqA1 and DRD4-7R) and Executive Function: Their Interaction with Obesity	6	6	6	6	6	6	5	6	6	6	6	65	Good quality
56	Markus and Capello, 2012	Contribution of the 5-HTTLPR gene by neuroticism on weight gain in male and female participants	6	6	6	5	6	6	5	6	6	6	6	64	Good quality
57	Chen *et al.*, 2012b	Association of genetic variants of glutamate decarboxylase 2 and the dopamine D2 receptor with obesity in Taiwanese subjects	6	6	6	6	6	6	5	6	6	6	6	65	Good quality
58	Winkler *et al.*, 2012	TaqIA variant in dopamine D2 receptor gene complicates weight maintenance in younger obese patients	6	6	6	6	6	6	5	6	6	6	6	65	Good quality
59	Peralta-Leal *et al.*, 2012	Association of a serotonin transporter gene (SLC6A4) 5-HTTLPR variant with body mass index categories but not type 2 diabetes mellitus in Mexicans	6	6	6	6	6	6	5	6	6	6	6	65	Good quality
60	Chen *et al.*, 2012a	Correlation of the Taq1 dopamine D2 receptor gene and percent body fat in obese and screened control subjects: A preliminary report	6	6	6	6	6	6	5	6	6	6	6	65	Good quality
61	Cameron *et al.*, 2012	The TaqIA RFLP is associated with attenuated intervention-induced body weight loss and increased carbohydrate intake in post-menopausal obese women	6	6	6	6	6	6	5	6	6	6	6	65	Good quality
62	Suriyaprom *et al.*, 2012	Association of HTTLPR and 5-HT$_2$A T102C variants with smoking characteristics and anthropometric profiles of Thai males	6	6	6	6	6	6	5	6	6	6	6	65	Good quality
63	Markus and Capello, 2012	Contribution of the 5-HTTLPR gene by neuroticism on weight gain in male and female participants	6	6	6	5	6	6	5	6	6	6	6	64	Good quality
64	Hill *et al.*, 2012	Catechol-O-methyltransferase (COMT) single nucleotide variants and haplotypes are not major risk factors for polycystic ovary syndrome	6	6	6	6	6	6	5	6	6	6	6	65	Good quality
65	Shinozaki *et al.*, 2012	Investigation of serotonin transporter gene (SLC6A4) by child abuse history interaction with body mass index and diabetes mellitus of White female depressed psychiatric inpatients	6	6	6	6	6	6	5	6	6	6	6	65	Good quality
66	Thaler *et al.*, 2012	Epistatic interactions implicating dopaminergic genes in bulimia nervosa (BN): Relationships to eating- and personality-related psychopathology	6	6	6	6	6	6	5	6	6	6	6	65	Good quality
67	Wallmeier *et al.*, 2013	Genetic modulation of the serotonergic pathway: Influence on weight reduction and weight maintenance	6	6	6	6	6	6	5	6	6	6	6	65	Good quality
68	Shinozaki *et al.*,	"Diminished" association between the serotonin transporter	6	6	6	6	6	6	5	6	6	6	6	65	Good quality

#	Study	Title	1	2	3	4	5	6	7	8	9	10	11	Total	Q-Genie classification
	2013	linked variant (5HTTLPR) and body mass index in a large psychiatric sample													
69	Carpenter *et al.*, 2013	Association of Dopamine D2 Receptor and Leptin Receptor Genes with Clinically Severe Obesity	6	6	6	6	6	6	5	6	6	6	6	65	Good quality
70	Roth *et al.*, 2013	Association analyses for dopamine receptor gene variants and weight status in a longitudinal analysis in obese children before and after lifestyle intervention	7	7	6	7	7	6	6	7	6	6	7	72	Good quality
71	Sikora *et al.*, 2013	Correlations between variants in genes coding elements of dopaminergic pathways and body mass index in overweight and obese women	6	6	5	7	7	4	4	6	6	6	7	64	Good quality
72	Capello *et al.*, 2014a	Differential influence of the 5-HTTLPR genotype, neuroticism and real-life acute stress exposure on appetite and energy intake	6	6	6	7	5	5	4	6	6	6	5	62	Good quality
73	Wang *et al.*, 2014	No Effect on Body Dissatisfaction of an Interaction between 5-HTTLPR Genotype and Neuroticism in a Young Adult Korean Population	6	6	6	7	5	5	5	6	6	6	5	63	Good quality
74	Hursel *et al.*, 2014	The Role of Catechol-O-Methyl Transferase Val(108/158)Met Variant (rs4680) in the Effect of Green Tea on Resting Energy Expenditure and Fat Oxidation: A Pilot Study	6	6	6	7	5	6	4	6	6	6	5	63	Good quality
75	Valomon *et al.*, 2014	Genetic variants of DAT1 and COMT differentially associate with actigraphy-derived sleep-wake cycles in young adults	6	6	6	7	5	5	5	6	6	6	5	63	Good quality
76	Capello *et al.*, 2014b	Effect of sub chronic tryptophan supplementation on stress-induced cortisol and appetite in subjects differing in 5-HTTLPR genotype and trait neuroticism	6	6	6	7	5	5	4	6	6	6	5	62	Good quality
77	Athanasoulia *et al.*, 2014	The effect of the ANKK1/DRD2 Taq1A variant on weight changes of dopaminergic treatment in prolactinomas	6	6	6	7	7	5	4	6	6	6	7	66	Good quality
78	Rob Markus *et al.*, 2014	Sucrose preload reduces snacking after mild mental stress in healthy participants as a function of 5-hydroxytryptamine transporter gene promoter variant	6	6	6	7	5	5	4	6	6	6	5	62	Good quality
79	Hameed *et al.*, 2015	Genetic association analysis of serotonin transporter variant (5-HTTLPR) with type 2 diabetes patients of Pakistani population	6	6	6	7	6	5	5	6	6	6	6	65	Good quality
80	Uzun *et al.*, 2015	Association of VNTR variants in DRD4, 5-HTT and DAT1 genes with obesity	7	6	6	7	7	5	5	6	6	6	7	68	Good quality

#	Study	Title	1	2	3	4	5	6	7	8	9	10	11	Total	Q-Genie classification
81	Dias *et al.*, 2016	Association of variants in 5-HTT (SLC6A4) and MAOA genes with measures of obesity in young adults of Portuguese origin	7	6	6	7	7	5	5	6	6	6	7	68	Good quality
82	Yang *et al.*, 2014	Genome Wide Association Study: Searching for Genes Underlying Body Mass Index in the Chinese	7	7	6	7	7	6	7	7	6	6	7	73	Good quality
83	Kvaløy *et al.*, 2015	Genetic Effects on Longitudinal Changes from Healthy to Adverse Weight and Metabolic Status — The HUNT Study	7	7	6	7	7	6	7	7	6	6	7	73	Good quality
84	Borkowska *et al.*, 2015	Effect of the 5-HTTLPR variant on affective temperament, depression and body mass index in obesity	7	6	6	7	7	5	5	6	6	6	7	68	Good quality
85	Yokum *et al.*, 2015	Relation of the multilocus genetic composite reflecting high dopamine signaling capacity to future increases in BMI	7	7	6	7	7	6	6	7	6	6	7	72	Good quality
86	Yeh *et al.*, 2016	Food Cravings, Food Addiction, and a Dopamine-Resistant (DRD2 A1) Receptor Variant in Asian American College Students	7	6	6	7	6	4	4	6	6	6	7	65	Good quality
87	Jawinski *et al.*, 2016	Time to wake up: No impact of COMT Val158Met gene variation on circadian preferences, arousal regulation and sleep	7	6	6	7	4	5	6	6	6	6	5	64	Good quality
88	Hinderberger *et al.*, 2016	The effect of serum BDNF levels on central serotonin transporter availability in obese versus non-obese adults: A [11C]DASB positron emission tomography study	7	6	6	7	7	5	4	6	6	6	6	66	Good quality
89	Yadav *et al.*, 2016	Dopamine receptor D2 gene variant and interaction with the body mass index: A study among two tribal populations of Central India	7	6	5	7	7	3	5	6	6	6	7	65	Good quality
90	Bieliński *et al.*, 2017	Association between COMT Val158Met and DAT1 variants and depressive symptoms in the obese population	7	6	5	7	7	4	5	6	6	6	7	66	Good quality
91	González-Giraldo *et al.*, 2018	Two dopaminergic genes, DRD4 and SLC6A3, are associated with body mass index in a Colombian sample of young adults	7	6	5	7	7	4	5	6	6	6	7	66	Good quality
92	Pedram *et al.*, 2017	Two novel candidate genes identified in adults from the Newfoundland population with addictive tendencies towards food	7	6	6	7	7	4	6	6	6	6	6	67	Good quality
93	Schepers and Markus, 2017	The interaction between 5-HTTLPR genotype and ruminative thinking on BMI	7	6	5	7	7	4	4	6	6	6	6	64	Good quality
94	Lek *et al.*, 2018	Association of dopamine receptor D2 gene (DRD2) Taq1 variants with eating behaviors and obesity among Chinese and Indian Malaysian university students	7	6	5	7	7	4	4	6	6	6	6	64	Good quality

#	Study	Title	1	2	3	4	5	6	7	8	9	10	11	Total	Q-Genie classification
95	Rivera-Iñiguez *et al.*, 2019	DRD2/ANKK1 TaqI A1 variant associates with overconsumption of unhealthy foods and biochemical abnormalities in a Mexican population	7	6	6	7	7	5	5	6	6	6	7	68	Good quality
96	Frank *et al.*, 2018	Dopamine D2 −141C Ins/Del and Taq1A variants, body mass index, and prediction error brain response	7	6	6	7	7	5	4	6	6	6	7	67	Good quality
97	Palacios *et al.*, 2018	Complete sequence of the ANKK1 gene in Mexican-Mestizo individuals with obesity, with or without binge eating disorder	7	6	6	7	7	4	5	6	6	6	7	67	Good quality
98	Pavlova *et al.*, 2019	Interrelation of the FTO rs9939609 SNP and the DAT1 rs27072 SNP with Body Mass Index and Degree of Obesity in the Population of Yakuts	7	6	5	7	7	4	5	6	6	6	7	66	Good quality
99	Ramos-Lopez *et al.*, 2019	Interactions between DRD2/ANKK1 TaqIA Variant and Dietary Factors Influence Plasma Triglyceride Concentrations in Diabetic Patients from Western Mexico: A Cross-sectional Study	7	6	6	7	6	6	5	6	6	6	6	67	Good quality
100	Mehri *et al.*, 2019	The Investigation of Functional Genetic Variation in COMT Gene Promoter (rs2020917 & rs2075507) in Iranian Patients with Breast Cancer	5	5	5	7	4	4	5	5	6	5	4	55	Good quality
101	Asadzadeh *et al.*, 2019	Investigation of the Association between 5-Hydroxytryptamine Transporter Gene-Linked Polymorphic Region with Type 2 Diabetes Mellitus, Obesity and Biochemical Profiles of Serum in Iranian Population	7	6	6	7	6	5	5	6	6	6	7	67	Good quality
102	Lim *et al.*, 2020	Influence of dopamine receptor gene on eating behaviour and obesity in Malaysia	7	6	6	7	7	6	6	6	6	6	7	70	Good quality
103	Galaviz-Hernández *et al.*, 2020	Association of the 5HTTLPR Variant with Obesity in Mexican Women with High Native American Ancestry	7	6	6	7	7	5	5	6	6	6	7	68	Good quality
104	Gassó *et al.*, 2020	Association study of candidate genes with obesity and metabolic traits in antipsychotic-treated patients with first-episode psychosis over a 2-year period	7	7	6	7	7	6	6	6	6	6	7	71	Good quality
105	Aliasghari *et al.*, 2021a	Genotypes of ANKK1 and DRD2 genes and risk of metabolic syndrome and its components: A cross-sectional study on Iranian women	7	6	6	7	7	5	6	6	6	6	7	69	Good quality
106	Aliasghari *et al.*, 2021	Associations of the ANKK1 and DRD2 gene variants with overweight, obesity and hedonic hunger among women from the Northwest of Iran	7	6	6	7	7	5	6	6	6	6	7	69	Good quality

#	Study	Title	1	2	3	4	5	6	7	8	9	10	11	Total	Q-Genie classification
107	Paderina *et al.*, 2021	Genetic Variants of 5-HT Receptors and Antipsychotic-Induced Metabolic Dysfunction in Patients with Schizophrenia	7	6	6	7	7	5	5	6	6	6	6	67	Good quality
108	Matsunaga *et al.*, 2021	A Genetic Variation in the Y Chromosome Among Modern Japanese Males Related to Several Physiological and Psychological Characteristics	6	6	7	7	7	5	7	6	6	6	6	69	Good quality
109	Beyer *et al.*, 2021	Higher BMI, but not obesity-related genetic variants, correlates with lower structural connectivity of the reward network in a population-based study	6	6	7	7	7	6	7	6	6	6	6	70	Good quality
110	Obregón *et al.*, 2022	Association of the dopamine D2 receptor rs1800497 variant with food addiction, food reinforcement, and eating behavior in Chilean adults	6	6	7	7	7	6	7	6	6	6	6	70	Good quality
111	Bednarova *et al.*, 2023	Association of HTTLPR, BDNF, and FTO Genetic Variants with Completed Suicide in Slovakia	6	6	7	7	7	6	7	6	6	6	6	70	Good quality
112	Arrue *et al.*, 2023	Multilocus Genetic Profile Reflecting Low Dopaminergic Signaling Is Directly Associated with Obesity and Cardiometabolic Disorders Due to Antipsychotic Treatment	6	7	7	7	6	6	7	7	6	7	7	73	Good quality
113	Daza-Hernández *et al.*, 2023	Analysis of Factors Associated with Outcomes of Bariatric Surgery: rs1800497 ANKK1, rs1799732 DRD2 Genetic Variants, Eating Behavior, Hedonic Hunger, and Depressive Symptoms	7	7	7	7	7	6	7	7	7	6	7	75	Good quality
114	Hidalgo Vira *et al.*, 2023	No association of the dopamine D2 receptor genetic bilocus score (rs1800497/rs1799732) on food addiction and food reinforcement in Chilean adults	6	6	7	7	7	6	7	6	6	6	6	70	Good quality
115	Losada-Casallas *et al.*, 2024	Body index mass not associated with DRD4, DAT1, BDNF, and COMT gene variants in young adults without depression or anxiety disorders	6	6	7	7	7	6	7	6	6	6	6	70	Good quality
116	Markus and Keulers, 2025	The serotonin gene 5-HTTLPR and brain food-reward responses during sadness: A mood-induction neuroimaging study	6	6	7	7	7	6	7	6	6	6	6	70	Good quality
117	Yatsuda *et al.*, 2025	Serotonin Transporter Gene Variants Predict Adherence to Weight Loss Programs Independently of Obesity-Related Genes	6	6	7	7	7	6	7	6	6	6	6	70	Good quality

List of questions: 1. Rationale for study; 2. Selection and definition of outcome of interest.; 3. Selection and comparability of comparison groups; 4. Technical classification of the exposure; 5. Non-technical classification of the exposure; 6. Other sources of bias; 7. Sample size and power; 8. A priori planning of analyses; 9. Statistical methods and control for confounding; 10. Testing of assumptions and inferences for genetic analyses; 11. Appropriateness of inferences drawn from results.

References

Aliasghari F, Mahdavi R, Barati M, Nazm SA, Yasari S, Bonyadi M and Jabbari M (2021a) Genotypes of ANKK1 and DRD2 genes and risk of metabolic syndrome and its components: A cross-sectional study on Iranian women. Obes Res Clin Pract 15:449–454.

Aliasghari F, Pirdehghan A, Aghamohammadzadeh N, Rashtchizadeh N, Azarfam P and Yaghmaei P (2021b) Associations of the ANKK1 and DRD2 gene variants with overweight, obesity and hedonic hunger among women from the Northwest of Iran. Eat Weight Disord 26:305–312.

Annerbrink K, Westberg L, Nilsson S, Rosmond R, Holm G and Eriksson E (2008) Catechol O-methyltransferase val158-met variant is associated with abdominal obesity and blood pressure in men. Metabolism 57:708–711.

Ariza M, Garolera M, Jurado MA, Garcia-Garcia I, Hernan I, Sánchez-Garre C, Vernet-Vernet M, Sender-Palacios MJ, Marques-Iturria I, Pueyo R *et al.* (2012) Dopamine genes (DRD2/ANKK1-TaqA1 and DRD4-7R) and executive function: Their interaction with obesity. PLoS One 7:e41482.

Arrue A, Olivas O, Erkoreka L, Alvarez FJ, Arnaiz A, Varela N, Bilbao A, Rodríguez JJ, Moreno-Calle MT and Gordo E (2023) Multilocus genetic profile reflecting low dopaminergic signaling is directly associated with obesity and cardiometabolic disorders due to antipsychotic treatment. Pharmaceutics 15:2134.

Asadzadeh A, Ghaheh HSS, Sholehvar F, Takhshid M and Naghizadeh MM (2019) Investigation of the association between 5-hydroxytryptamine transporter gene-linked polymorphic region with type 2 diabetes mellitus, obesity and biochemical profiles of serum in Iranian population. Avicenna J Med Biotechnol 11:239.

Athanasoulia AP, Sievers C, Uhr M, Ising M, Stalla GK and Schneider HJ (2014) The effect of the ANKK1/DRD2 Taq1A variant on weight changes of dopaminergic treatment in prolactinomas. Pituitary 17:240–245.

Azzato EM, Morton LM, Bergen AW, Wang SS, Chatterjee N, Kvale P, Yeager M, Hayes RB, Chanock SJ and Caporaso NE (2009) SLC6A3 and body mass index in the Prostate, Lung, Colorectal and Ovarian Cancer Screening Trial. BMC Med Genet 10:9.

Bah J, Westberg L, Baghaei F, Henningsson S, Rosmond R, Melke J, Holm G and Eriksson E (2010) Further exploration of the possible influence of variants in HTR2C and 5HTT on body weight. Metabolism 59:1156–1163.

Barnard ND, Noble EP, Ritchie T, Cohen J, Jenkins DJ, Turner-McGrievy G, Gloede L, Green AA and Ferdowsian H (2009) D2 dopamine receptor Taq1A variant, body weight, and dietary intake in type 2 diabetes. Nutrition 25:58–65.

Bednarova A, Habalova V, Iannaccone SF, Tkac I, Jarcuskova D, Krivosova M, Marcatili M and Hlavacova N (2023) Association of HTTLPR, BDNF, and FTO genetic variants with completed suicide in Slovakia. J Pers Med 13:501.

Beyer F, Zhang R, Scholz M, Wirkner K, Loeffler M, Stumvoll M, Villringer A and Witte AV (2021) Higher BMI, but not obesity-related genetic variants, correlates with lower structural connectivity of the reward network in a population-based study. Int J Obes (Lond) 45:491–501.

Bieliński M, Jaracz M, Lesiewska N, Tomaszewska M, Sikora M, Junik R, Kamińska A, Tretyn A and Borkowska A (2017) Association between COMT Val158Met and DAT1 variants and depressive symptoms in the obese population. Neuropsychiatr Dis Treat 13:2221–2229.

Blum K, Braverman ER, Wood RC, Gill J, Li C, Chen TJ, Taub M, Montgomery AR, Sheridan PJ and Cull JG (1996) Increased prevalence of the TaqI A1 allele of the dopamine receptor gene (DRD2) in obesity with comorbid substance use disorder: A preliminary report. Pharmacogenetics 6:297–305.

Borkowska A, Bieliński M, Szczęsny W, Szwed K, Tomaszewska M, Kałwa A, Lesiewska N, Junik R, Gołębiewski M, Sikora M *et al*. (2015) Effect of the 5-HTTLPR variant on affective temperament, depression and body mass index in obesity. J Affect Disord 184:193–197.

Brummett BH, Boyle SH, Siegler IC, Zuchner S, Ashley-Koch A and Williams RB (2008) Lipid levels are associated with a regulatory variant of the monoamine oxidase-A gene promoter (MAOA-uVNTR). Med Sci Monit 14:CR57–CR61.

Camarena B, Santiago H, Aguilar A, Ruvinskis E, González-Barranco J and Nicolini H (2004) Family-based association study between the monoamine oxidase A gene and obesity: Implications for psychopharmacogenetic studies. Neuropsychobiology 49:126–129.

Cameron JD, Riou MÈ, Tesson F, Goldfield GS, Rabasa-Lhoret R, Brochu M and Doucet É (2013) The TaqIA RFLP is associated with attenuated intervention-induced body weight loss and increased carbohydrate intake in post-menopausal obese women. Appetite 60:111–116.

Capello AE and Markus CR (2014a) Differential influence of the 5-HTTLPR genotype, neuroticism and real-life acute stress exposure on appetite and energy intake. Appetite 77:83–93.

Capello AE and Markus CR (2014b) Effect of sub chronic tryptophan supplementation on stress-induced cortisol and appetite in subjects differing in 5-HTTLPR genotype and trait neuroticism. Psychoneuroendocrinology 45:96–107.

Carpenter CL, Wong AM, Li Z, Noble EP and Heber D (2013) Association of dopamine D2 receptor and leptin receptor genes with clinically severe obesity. Obesity (Silver Spring) 21:E467–E473.

Chen TJH, Blum K, Mathews D, Fisher L, Schnautz N and Braverman ER (2007) Reviewing the role of putative candidate genes in “Neurobesigenics”, a clinical subtype of Reward Deficiency Syndrome (RDS). Gene Ther Mol Biol 11:61–74.

Chen AL, Blum K, Chen TJ, Giordano J, Downs BW, Han D, Barh D and Braverman ER (2012a) Correlation of the Taq1 dopamine D2 receptor gene and percent body fat in obese and screened control subjects: A preliminary report. Food Funct 3:40–48

Chen KC, Lin YC, Chao WC, Chung HK, Chi SS, Liu WS and Wu WT (2012b) Association of genetic variants of glutamate decarboxylase 2 and the dopamine D2 receptor with obesity in Taiwanese subjects. Ann Saudi Med 32:121–126.

Comings DE, Flanagan SD, Dietz G, Muhleman D, Knell E and Gysin R (1993) The dopamine D2 receptor (DRD2) as a major gene in obesity and height. Biochem Med Metab Biol 50:176–185.

Correia CT, Almeida JP, Santos PE, Sequeira AF, Marques CE, Miguel TS, Abreu RL, Oliveira GG and Vicente AM (2010) Pharmacogenetics of risperidone therapy in autism: Association analysis of eight candidate genes with drug efficacy and adverse drug reactions. Pharmacogenomics J 10:418–430.

Cribb AE, Joy Knight M, Guernsey J, Dryer D, Hender K, Shawwa A, Tesch M and Saleh TM (2011) CYP17, catechol-O-methyltransferase, and glutathione transferase M1 genetic variants, lifestyle factors, and breast cancer risk in women on Prince Edward Island. Breast J 17:24–31.

Davis C, Levitan RD, Kaplan AS, Carter J, Reid C, Curtis C, Patte K, Hwang R and Kennedy JL (2008) Reward sensitivity and the D2 dopamine receptor gene: A case-control study of binge eating disorder. Prog Neuropsychopharmacol Biol Psychiatry 32:620–628.

Davis CA, Levitan RD, Reid C, Carter JC, Kaplan AS, Patte KA, King N, Curtis C and Kennedy JL (2009) Dopamine for “wanting” and opioids for "liking": A comparison of obese adults with and without binge eating. Obesity (Silver Spring) 17:1220–1225.

Daza-Hernández S, Pérez-Luque E, Martínez-Cordero C, Figueroa-Vega N, Cardona-Alvarado MI and Muñoz-Montes N (2023) Analysis of factors associated with outcomes of bariatric surgery: rs1800497 ANKK1, rs1799732 DRD2 genetic variants, eating behavior, hedonic hunger, and depressive symptoms. J Gastrointest Surg 27:1778-1784.

Dias H, Muc M, Padez C and Manco L (2016) Association of variants in 5-HTT (SLC6A4) and MAOA genes with measures of obesity in young adults of Portuguese origin. Arch Physiol Biochem 122:8–13.

Ducci F, Newman TK, Funt S, Brown GL, Virkkunen M and Goldman D (2006) A functional variant in the MAOA gene promoter (MAOA-LPR) predicts central dopamine function and body mass index. Mol Psychiatry 11:858–866.

Eisenberg DT, Campbell B, Gray PB and Sorenson MD (2008) Dopamine receptor genetic variants and body composition in undernourished pastoralists: An exploration of nutrition indices among nomadic and recently settled Ariaal men of northern Kenya. BMC Evol Biol 8:173.

Epstein LH, Jaroni JL, Paluch RA, Leddy JJ, Vahue HE, Hawk L, Wileyto EP, Shields PG and Lerman C (2002) Dopamine transporter genotype as a risk factor for obesity in African-American smokers. Obes Res 10:1232–1240.

Epstein LH, Wright SM, Paluch RA, Leddy JJ, Hawk LW Jr, Jaroni JL, Saad FG, Crystal-Mansour S, Shields PG and Lerman C (2004) Relation between food reinforcement and dopamine genotypes and its effect on food intake in smokers. Am J Clin Nutr 80:82–88.

Epstein LH, Temple JL, Neaderhiser BJ, Salis RJ, Erbe RW and Leddy JJ (2007) Food reinforcement, the dopamine D2 receptor genotype, and energy intake in obese and nonobese humans. Behav Neurosci 121:877–886.

Epstein LH, Dearing KK and Erbe RW (2011) Parent-child concordance of Taq1 A1 allele predicts similarity of parent-child weight loss in behavioral family-based treatment programs. Appetite 55:363–366.

Fang YJ, Thomas GN, Xu ZL, Fang JQ, Critchley JA and Tomlinson B (2005) An affected pedigree member analysis of linkage between the dopamine D2 receptor gene TaqI variant and obesity and hypertension. Int J Cardiol 102:111–116.

Frank GKW, Shott ME, DeGuzman MC and Smolen A (2018) Dopamine D2 -141C Ins/Del and Taq1A variants, body mass index, and prediction error brain response. Transl Psychiatry 8:102.

Galaviz-Hernández C, Lazalde-Ramos BP, Martínez-Cortés G, Rangel-Villalobos H, Martínez-Aguilar G, Leal-Ugarte E, Peralta-Leal V *et al.* (2020) Association of the 5HTTLPR variant with obesity in Mexican women with high Native American ancestry. Genet Test Mol Biomarkers 24:754–758.

Gallicchio L, Chang HH, Christo DK, Thuita L, Huang HY, Strickland P, Ruczinski I, Clipp S and Helzlsouer KJ (2009) Single nucleotide variants in obesity-related genes and all-cause and cause-specific mortality: A prospective cohort study. BMC Med Genet 10:103.

Gassó P, Arnaiz JA, Mas S, Lafuente A, Bioque M, Cuesta MJ, Díaz-Caneja CM, García C, Lobo A, González-Pinto A *et al.* (2020) Association study of candidate genes with obesity and metabolic traits in antipsychotic-treated patients with first-episode psychosis over a 2-year period. J Psychopharmacol 34:514–523.

González-Giraldo Y, Trujillo ML and Forero DA (2018) Two dopaminergic genes, DRD4 and SLC6A3, are associated with body mass index in a Colombian sample of young adults. Arch Physiol Biochem 124:330–334.

Gorai I, Inada M, Morinaga H, Uchiyama Y, Yamauchi H, Hirahara F and Chaki O (2007) CYP17 and COMT gene variants can influence bone directly, or indirectly through their effects on endogenous sex steroids, in postmenopausal Japanese women. Bone 40:28–36.

Hameed A, Ajmal M, Nasir M and Ismail M (2015) Genetic association analysis of serotonin transporter variant (5-HTTLPR) with type 2 diabetes patients of Pakistani population. Diabetes Res Clin Pract 108:67–71.

Hidalgo Vira N, Oyarce K, Valladares Vega M, Goldfield GS, Guzmán-Gutiérrez E and Obregón AM (2023) No association of the dopamine D2 receptor genetic bilocus score (rs1800497/rs1799732) on food addiction and food reinforcement in Chilean adults. Front Behav Neurosci 17:1067384.

Hill LD, Ewens KG, Maher BS, York TP, Legro RS, Dunaif A and Strauss JF 3rd (2012) Catechol-O-methyltransferase (COMT) single nucleotide variants and haplotypes are not major risk factors for polycystic ovary syndrome. Mol Cell Endocrinol 350:72–77.

Hinderberger P, Rullmann M, Drabe M, Luthardt J, Becker GA, Blüher M, Regenthal R, Sabri O and Hesse S (2016) The effect of serum BDNF levels on central serotonin transporter availability in obese versus non-obese adults: A [(11)C]DASB positron emission tomography study. Neuropharmacology 110:530–536.

Hong CC, Thompson HJ, Jiang C, Hammond GL, Tritchler D, Yaffe M and Boyd NF (2003) Val158Met variant in catechol-O-methyltransferase gene associated with risk factors for breast cancer. Cancer Epidemiol Biomarkers Prev 12:838–847.

Hursel R, Janssens PL, Bouwman FG, Mariman EC and Westerterp-Plantenga MS (2014) The role of catechol-O-methyl transferase Val(108/158)Met variant (rs4680) in the effect of green tea on resting energy expenditure and fat oxidation: A pilot study. PLoS One 9:e106220.

Iordanidou M, Tavridou A, Petridis I, Arvanitidis KI, Christakidis D, Vargemezis V and Manolopoulos VG (2010) The serotonin transporter promoter variant (5-HTTLPR) is associated with type 2 diabetes. Clin Chim Acta 411:167–171.

Jawinski P, Tegelkamp S, Sander C, Häntzsch M, Huang J, Mauche N, Scholz M, Spada J, Ulke C, Burkhardt R *et al.* (2016) Time to wake up: No impact of COMT Val158Met gene variation on circadian preferences, arousal regulation and sleep. Chronobiol Int 33:893–905.

Justenhoven C, Hamann U, Schubert F, Zapatka M, Pierl CB, Rabstein S, Selinski S, Mueller T, Ickstadt K, Gilbert M *et al.* (2008) Breast cancer: A candidate gene approach across the estrogen metabolic pathway. Breast Cancer Res Treat 137:49.

Kocabaş NA, Sardaş S, Cholerton S, Daly AK and Karakaya AE (2002) Cytochrome P450 CYP1B1 and catechol O-methyltransferase (COMT) genetic variants and breast cancer susceptibility in a Turkish population. Arch Toxicol 76:643–649.

Kocabaş NA, Sardaş S and Karakaya AE (2005) Variants related to estrogen and xenobiotic metabolism in healthy Turkish women. Arch Med Res 36:19–23.

Kring SI, Werge T, Holst C, Toubro S, Astrup A, Hansen T, Pedersen O and Sørensen TI (2009) Variants of serotonin receptor 2A and 2C genes and COMT in relation to obesity and type 2 diabetes. PLoS One 4:e6696.

Kvaløy K, Holmen J, Hveem K and Holmen TL (2015) Genetic effects on longitudinal changes from healthy to adverse weight and metabolic status—the HUNT study. PLoS One 10:e0139632.

Lan MY, Chang YY, Chen WH, Kao YF, Lin HS and Liu JS (2009) Serotonin transporter gene promoter variant is associated with body mass index and obesity in non-elderly stroke patients. J Endocrinol Invest 32:119–122.

Lavigne JA, Helzlsouer KJ, Huang HY, Strickland PT, Bell DA, Selmin O, Watson MA, Hoffman S, Comstock GW and Yager JD (1997) An association between the allele coding for a low activity variant of catechol-O-methyltransferase and the risk for breast cancer. Cancer Res 57:5493–5497.

Lee HY, Kim DJ, Lee HJ, Choi JE and Kim YK (2009) No association of serotonin transporter variant (5-HTTVNTR and 5-HTTLPR) with characteristics and treatment response to atypical antipsychotic agents in schizophrenic patients. Prog Neuropsychopharmacol Biol Psychiatry 33:276–280.

Lek FY, Ong HH and Say YH (2018) Association of dopamine receptor D2 gene (DRD2) Taq1 variants with eating behaviors and obesity among Chinese and Indian Malaysian university students. Asia Pac J Clin Nutr 27:707–717.

Levitan RD, Kaplan AS, Davis C, Lam RW and Kennedy JL (2010) A season-of-birth/DRD4 interaction predicts maximal body mass index in women with bulimia nervosa. Neuropsychopharmacology 35:1729–1733.

Lim ZM, Chie QT and Teh LK (2020) Influence of dopamine receptor gene on eating behaviour and obesity in Malaysia. Meta Gene 25:100736.

Lloret Linares C, Hajj A, Poitou C, Simoneau G, Clement K, Laplanche JL, Lépine JP, Bergmann JF, Mouly S and Peoc'h K (2011) Pilot study examining the frequency of several gene variants involved in morphine pharmacodynamics and pharmacokinetics in a morbidly obese population. Obes Surg 21:1257–1264.

Losada-Casallas K, Cepeda-Leal I, Ruiz N, Muñoz-Ospina B and Ortega-Avila G (2024) Body index mass not associated with DRD4, DAT1, BDNF, and COMT gene variants in young adults without depression or anxiety disorders. Genet Mol Res 23:1–10.

Markus CR and Capello AEM (2012) Contribution of the 5-HTTLPR gene by neuroticism on weight gain in male and female participants. Psychiatr Genet 22:279–285.

Markus CR and Keulers EHH (2025) The serotonin gene 5-HTTLPR and brain food-reward responses during sadness: A mood-induction neuroimaging study. J Affect Disord 384:1–11.

Matsunaga M, Ohtsubo Y, Masuda T, Noguchi Y, Yamasue H and Ishii K (2021) A genetic variation in the Y chromosome among modern Japanese males related to several physiological and psychological characteristics. Front Behav Neurosci 15:774879.

Mehri F, Tahmasebi Fard Z and Ghoraeian P (2019) The investigation of functional genetic variation in COMT gene promoter (rs2020917 & rs2075507) in Iranian patients with breast cancer. Int J Cancer Manag 12:e92008.

Mergen H, Karaaslan C, Mergen M, Deniz Ozsoy E and Ozata M (2007) LEPR, ADBR3, IRS-1 and 5-HTT genes variants do not associate with obesity. Endocr J 54:89-94.

Millikan RC, Pittman GS, Tse CK, Duell E, Newman B, Savitz D, Moorman PG, Boissy RJ and Bell DA (1998) Catechol-O-methyltransferase and breast cancer risk. Carcinogenesis 19:1943–1947.

Mitrunen K, Jourenkova N, Kataja V, Eskelinen M, Kosma VM, Benhamou S, Kang D, Vainio H, Uusitupa M and Hirvonen A (2001) Polymorphic catechol-O-methyltransferase gene and breast cancer risk. Cancer Epidemiol Biomarkers Prev 10:635–640.

Morton LM, Wang SS, Bergen AW, Chatterjee N, Kvale P, Welch R, Yeager M, Hayes RB, Chanock SJ and Caporaso NE (2006) DRD2 genetic variation in relation to smoking and obesity in the Prostate, Lung, Colorectal, and Ovarian Cancer Screening Trial. Pharmacogenet Genomics 16:901–910.

Munafò MR, Murphy MF and Johnstone EC (2006) Smoking cessation, weight gain, and DRD4−521 genotype. Am J Med Genet B Neuropsychiatr Genet 141:398–402.

Need AC, Ahmadi KR, Spector TD and Goldstein DB (2006) Obesity is associated with genetic variants that alter dopamine availability. Ann Hum Genet 70:293–303.

Nisoli E, Brunani A, Borgomainerio E, Tonello C, Dioni L, Briscini L, Redaelli G, Molinari E, Cavagnini F and Carruba MO (2007) D2 dopamine receptor (DRD2) gene Taq1A variant and the eating-related psychological traits in eating disorders (anorexia nervosa and bulimia) and obesity. Eat Weight Disord 12:91–96.

Noble EP, Noble RE, Ritchie T, Syndulko K, Bohlman MC, Noble LA, Zhang Y, Sparkes RS and Grandy DK (1994) D2 dopamine receptor gene and obesity. Int J Eat Disord 15:205–217.

Obregón AM, Oyarce K, García-Robles MA, Valladares M, Pettinelli P and Goldfield GS (2022) Association of the dopamine D2 receptor rs1800497 variant with food addiction, food reinforcement, and eating behavior in Chilean adults. Eat Weight Disord 27:215–224.

Paderina DZ, Boiko AS, Pozhidaev IV, Bocharova AV, Mednova IA, Fedorenko OY, Kornetova EG, Loonen AJM, Semke AV, Bokhan NA *et al.* (2021) Genetic variants of 5-HT receptors and antipsychotic-induced metabolic dysfunction in patients with schizophrenia. J Pers Med 11:181.

Palacios A, Canto P, Tejeda ME, Stephano S, Luján H, García-García E, Rojano-Mejía D and Méndez JP (2018) Complete sequence of the ANKK1 gene in Mexican-Mestizo individuals with obesity, with or without binge eating disorder. Eur Psychiatry 54:59–64.

Pavlova NI, Kurtanov KA, Diakonova AT, Solovyeva NA, Sydykova LA, Aleksandrova TN and Solovyeva YA (2019) Interrelation of the FTO rs9939609 SNP and the DAT1 rs27072 SNP with body mass index and degree of obesity in the population of Yakuts. Int J Biomed 9:210–215.

Pedram P, Zhai G, Gulliver W, Zhang H and Sun G (2017) Two novel candidate genes identified in adults from the Newfoundland population with addictive tendencies towards food. Appetite 115:71–79.

Peralta-Leal V, Leal-Ugarte E, Meza-Espinoza JP, Dávalos-Rodríguez IP, Bocanegra-Alonso A, Acosta-González RI, Gonzales E, Nair S and Durán-González J (2012) Association of a serotonin transporter gene (SLC6A4) 5-HTTLPR variant with body mass index categories but not type 2 diabetes mellitus in Mexicans. Genet Mol Biol 35:589–593.

Poston WS 2nd, Ericsson M, Linder J, Haddock CK, Hanis CL, Nilsson T, Aström M and Foreyt JP (1998) D4 dopamine receptor gene exon III variant and obesity risk. Eat Weight Disord 3:71–77.

Ramos-Lopez O, Mejia-Godoy R, Frías-Delgadillo KJ, Torres-Valadez R, Flores-García A, Sánchez-Enríquez S, Aguiar-García P, Martínez-López E and Zepeda-Carrillo EA (2019) Interactions between DRD2/ANKK1 TaqIA variant and dietary factors influence plasma triglyceride concentrations in diabetic patients from Western Mexico: A cross-sectional study. Nutrients 11:2863.

Rivera-Iñiguez I, Panduro A, Ramos-Lopez O, Villaseñor-Bayardo SJ and Roman S (2019) DRD2/ANKK1 TaqI A1 variant associates with overconsumption of unhealthy foods and biochemical abnormalities in a Mexican population. Eat Weight Disord 24:835–844.

Roth CL, Hinney A, Schur EA, Elfers CT and Reinehr T (2013) Association analyses for dopamine receptor gene variants and weight status in a longitudinal analysis in obese children before and after lifestyle intervention. BMC Pediatr 13:197.

Schepers R and Markus CR (2017) The interaction between 5-HTTLPR genotype and ruminative thinking on BMI. Br J Nutr 118:629–637.

Shinozaki G, Romanowicz M, Kung S, Rundell J and Mrazek D (2012) Investigation of serotonin transporter gene (SLC6A4) by child abuse history interaction with body mass index and diabetes mellitus of White female depressed psychiatric inpatients. Psychiatr Genet 22:109–114.

Shinozaki G, Kumar Y, Rosen BH, Rundell JR, Mrazek DA and Kung S (2013) “Diminished” association between the serotonin transporter linked variant (5HTTLPR) and body mass index in a large psychiatric sample. J Affect Disord 151:397-400.

Sikora M, Gese A, Czypicki R, Gąsior M, Tretyn A, Chojnowski J, Bieliński M, Jaracz M, Kamińska A, Junik R, Borkowska A (2013) Correlations between variants in genes coding elements of dopaminergic pathways and body mass index in overweight and obese women. Endokrynol Pol 64:101–107.

Sookoian S, Gianotti TF, Gemma C, Burgueno A and Pirola CJ (2008) Contribution of the functional 5-HTTLPR variant of the SLC6A4 gene to obesity risk in male adults. Obesity 16:488–491.

Southon A, Walder K, Sanigorski AM, Zimmet P, Nicholson GC, Kotowicz MA and Collier G (2003) The Taq IA and Ser311 Cys variants in the dopamine D2 receptor gene and obesity. Diabetes Nutr Metab 16:72–76.

Spitz MR, Detry MA, Pillow P, Hu YY, Amos CI, Hong WK and Wu X (2000) Variant alleles of the D2 dopamine receptor gene and obesity. Nutr Res 20:371–380.

Suriyaprom K, Phonrat B, Chuensumran U, Tungtrongchitr A and Tungtrongchitr R (2012) Association of HTTLPR and 5-HT$_2$A T102C variants with smoking characteristics and anthropometric profiles of Thai males. Genet Mol Res 11:4360–4369.

Thaler L, Groleau P, Badawi G, Sycz L, Zeramdini N, Too A, Israel M, Joober R, Bruce KR and Steiger H (2012) Epistatic interactions implicating dopaminergic genes in bulimia nervosa (BN): Relationships to eating-and personality-related psychopathology. Prog Neuropsychopharmacol Biol Psychiatry 39:120–128.

Thomas GN, Tomlinson B and Critchley JA (2000) Modulation of blood pressure and obesity with the dopamine D2 receptor gene TaqI variant. Hypertension 36:177–182.

Thomas GN, Critchley JAJH, Tomlinson B, Cockram CS and Chan JCN (2001) Relationships between the TaqI variant of the dopamine D2 receptor and blood pressure in hyperglycaemic and normoglycaemic Chinese subjects. Clin Endocrinol 55:605–611.

Thompson PA, Shields PG, Freudenheim JL, Stone A, Vena JE, Marshall JR, Graham S, Laughlin R, Nemoto T, Kadlubar FF *et al.* (1998) Genetic variants in catechol-O-methyltransferase, menopausal status, and breast cancer risk. Cancer Res 58:2107–2110.

Tsuboi H, Sakakibara H, Yamakawa-Kobayashi K, Tatsumi A, Inamori T, Hamamoto R, Suzuki A and Shimoi K (2011) Val1483Ile variant in the fatty acid synthase gene was associated with depressive symptoms under the influence of psychological stress. J Affect Disord 134:448–452.

Tworoger SS, Chubak J, Aiello EJ, Yasui Y, Ulrich CM, Farin FM, Stapleton PL, Irwin ML, Potter JD, Schwartz RS *et al.* (2004) The effect of CYP19 and COMT variants on exercise-induced fat loss in postmenopausal women. Obes Res 12:972–981.

Uzun M, Saglar E, Kucukyildirim S, Erdem B, Unlu H and Mergen H (2015) Association of VNTR variants in DRD4, 5-HTT and DAT1 genes with obesity. Arch Physiol Biochem 121:75–79.

Valomon A, Holst SC, Bachmann V, Viola AU, Schmidt C, Zürcher J, Berger W, Cajochen C and Landolt HP (2014) Genetic variants of DAT1 and COMT differentially associate with actigraphy-derived sleep-wake cycles in young adults. Chronobiol Int 31:705–714.

Wallmeier D, Winkler JK, Fleming T, Woehning A, Huennemeyer K, Roeder E, Nawroth PP, Friederich HC, Wolfrum C, Schultz JH *et al.* (2013) Genetic modulation of the serotonergic pathway: Influence on weight reduction and weight maintenance. Genes Nutr 8:601–610.

Wang SS, Morton LM, Bergen AW, Lan EZ, Chatterjee N, Kvale P, Hayes RB, Chanock SJ and Caporaso NE (2007) Genetic variation in catechol-O-methyltransferase (COMT) and obesity in the prostate, lung, colorectal, and ovarian (PLCO) cancer screening trial. Hum Genet 122:41–49.

Wang SK, Lee YH, Kim JL and Chee IS (2014) No effect on body dissatisfaction of an interaction between 5-HTTLPR genotype and neuroticism in a young adult Korean population. Clin Psychopharmacol Neurosci 12:229–234.

Winkler JK, Woehning A, Schultz JH, Brune M, Beaton N, Challa TD, Minkova S, Roeder E, Nawroth PP, Friederich HC, Wolfrum C and Rudofsky G (2012) TaqIA variant in dopamine D2 receptor gene complicates weight maintenance in younger obese patients. Nutrition 28:996–1001.

Witte AV, Jansen S, Schirmacher A, Young P and Flöel A (2010) COMT Val158Met variant modulates cognitive effects of dietary intervention. Front Aging Neurosci 2:146.

Yadav S, Devi NM, Singh HJ and Saraswathy KN (2016) Dopamine receptor D2 gene variant and interaction with the body mass index: A study among two tribal populations of Central India. Gene Rep 4:269–271.

Yang F, Chen XD, Tan LJ, Shen J, Li DY, Zhang F, Sha BY and Deng HW (2014) Genome wide association study: Searching for genes underlying body mass index in the Chinese. Biomed Environ Sci 27:360–370.

Yatsuda M, Furou M, Kamachi K, Sakamoto K, Shoji K, Ishihara O and Kagawa Y (2025) Serotonin transporter gene variants predict adherence to weight loss programs independently of obesity-related genes. Nutrients 17:1094.

Yeh J, Trang A, Henning SM, Wilhalme H, Carpenter C, Heber D and Li Z (2016) Food cravings, food addiction, and a dopamine-resistant (DRD2 A1) receptor variant in Asian American college students. Asia Pac J Clin Nutr 25:424–429.

Yim DS, Parkb SK, Yoo KY, Yoon KS, Chung HH, Kang HL, Ahn SH, Noh DY, Choe KJ, Jang IJ *et al.* (2001) Relationship between the Val158Met variant of catechol O-methyl transferase and breast cancer. Pharmacogenetics 11:279–286.

Yokum S, Marti CN, Smolen A and Stice E (2015) Relation of the multilocus genetic composite reflecting high dopamine signaling capacity to future increases in BMI. Appetite 87:38–45.

Zhang ZJ, Yao ZJ, Zhang XB, Chen JF, Sun J, Yao H, Hou G and Zhang XB (2003) No association of antipsychotic agent-induced weight gain with a DA receptor gene variant and therapeutic response. Acta Pharmacol Sin 24:235–240.
